# Subjective well-being across the life course among non-industrialized populations

**DOI:** 10.1126/sciadv.ado0952

**Published:** 2024-10-23

**Authors:** Michael Gurven, Yoann Buoro, Daniel Eid Rodriguez, Katherine Sayre, Benjamin Trumble, Aili Pyhälä, Hillard Kaplan, Arild Angelsen, Jonathan Stieglitz, Victoria Reyes-García

**Affiliations:** ^1^ Department of Anthropology, University of California Santa Barbara, Santa Barbara, CA, USA.; ^2^ Universidad de San Simon, Cochabamba, Bolivia.; ^3^ School for Human Evolution and Social Change, Center for Evolution and Medicine, Arizona State University, Tempe, AZ, USA.; ^4^ Global Development Studies, Faculty of Social Sciences, University of Helsinki, Helsinki, Finland.; ^5^ Economic Science Institute, Argyros School of Business and Economics, Chapman University, Orange, CA, USA.; ^6^ School of Economics and Business, Norwegian University of Life Sciences (NMBU), Ås, Norway.; ^7^ Center for International Forestry Research (CIFOR), Bogor, Indonesia.; ^8^ Department of Social and Behavioral Sciences, Toulouse School of Economics, Université Toulouse 1 Capitole, Toulouse, France.; ^9^ Institut de Ciència i Tecnologia Ambientals, Universitat Autònoma de Barcelona, Barcelona, Spain.; ^10^ Institució Catalana de Recerca i Estudis Avançats, Barcelona, Spain.; ^11^ Departament d’Antropologia Social i Cultural, Universitat Autònoma de Barcelona, Bellaterra, Spain.

## Abstract

Subjective well-being (SWB) is often described as being U-shaped over adulthood, declining to a midlife slump and then improving thereafter. Improved SWB in later adulthood has been considered a paradox given age-related declines in health and social losses. While SWB has mostly been studied in high-income countries, it remains largely unexplored in rural subsistence populations lacking formal institutions that reliably promote social welfare. Here, we evaluate the age profile of SWB among three small-scale subsistence societies (*n* = 468; study 1), forest users from 23 low-income countries (*n* = 6987; study 2), and Tsimane’ horticulturalists (*n* = 1872; study 3). Across multiple specifications, we find variability in SWB age profiles. In some cases, we find no age-related differences in SWB or even inverted U-shapes. Adjusting for confounders reduces observed age effects. Our findings highlight variability in average well-being trajectories over the life course. Ensuring successful aging will require a greater focus on cultural and socioecological determinants of individual trajectories.

## Teaser

Subjective well-being in small-scale subsistence-oriented societies shows variable trajectories over adulthood.

## INTRODUCTION

Global increases in life expectancy, especially amid challenging socioeconomic circumstances, demand an improved understanding of well-being in mid- and late-life. One popular proposal is that happiness or well-being is “U-shaped” with age in human populations. Whether assessed as self-reports of life satisfaction, well-being, or happiness or from less subjective measures (e.g., “deaths of despair” including suicide, antidepressant prescriptions) (*1*, *2*), a U-shaped age curve refers to the tendency for proxies of well-being to first decline from teenage years to a nadir in middle adulthood (“midlife crisis,” 40 to 50 years). Where observed, the size of the dip in well-being in midlife is sometimes small but is equivalent in magnitude to the effects of unemployment or divorce (*3*). Following this midlife slump, well-being then increases appreciably. This increase, in the face of declining health and social losses, has been labeled a major paradox. Beyond age ~70 years, well-being tends to decline again [see, e.g., (*4*, *5*)], though age patterns in late adulthood are inconsistent across studies. Although evidence supporting a U-shape curve is ample [see (*3*, *6*, *7*)], concerns over analytical methods and sampling suggest mixed results, and no consensus (*8*–*10*).

The U-shape curve was first identified in United States and European datasets, though a recent review of 618 studies reports U-shapes between happiness and age across 145 countries (*6*, *11*), including several low-income countries. The U-shaped happiness-age curve is not only reported widely among humans but also among nonhuman primates, including chimpanzees and orangutans (*12*), which has led some U-shape proponents to conclude that “the existence of a midlife low in well-being is among the most striking, persistent and consistent patterns in the social sciences” [(*11*), p. 317]. However, the apes in the reference study lived in captivity or in sanctuaries, the caretakers who rated the apes came from high-income countries, and this single study of SWB in nonhuman primates has never been replicated.

Claims about any particular relationship between happiness and age are based inductively on empirical evidence. The U-shape curve has been revealed primarily as a statistical exercise, without a priori theory that predicts a particular shape, much less a universal species-wide or even taxonomic family-wide pattern common to all hominids. Post hoc reasoning is that a common U-shape in humans and great apes may be due to “similar age-related changes in brain structures associated with wellbeing” [(*12*), p. 2]. Alternatively, the U-shape could result from forecast errors over the life course, whereby an optimism bias coupled with unmet aspirations lowers people’s well-being by midlife, but expectations are abandoned or experienced with less regret during old age (*13*). As López Ulloa and colleagues (*9*) pointed out a decade ago in their summary of this controversial field spanning economics, psychology, and gerontology, multiple shapes have been identified, and a variety of post hoc theories can be invoked to account for any shape.

On the empirical side, identifying “pure” age effects of subjective well-being (SWB) over the life course is complicated. Age cannot be fully disentangled from period and cohort effects due to collinearity (i.e., since age = period − cohort, there is an age-period-cohort identification problem), which is only partially resolved through longitudinal study design (*14*). Much of the evidence for the U-shape relies on cross-sectional data, though the U-shape has been confirmed in several longitudinal studies [see, e.g., (*15*–*19*)], and after taking into account attrition due to higher mortality among unhappy participants (*18*). The decision about whether to control for important events that may change with age is also controversial (*20*–*22*), but statistical adjustments for marriage, divorce, job loss, having children, and other major life events associated with age often do not substantially alter the U-shape [but see (*23*)]. Inferences about the U-shape are sometimes robust to statistical model specification, though recent studies show how the U-shape can disappear when moving away from a reliance on quadratic age terms and when considering longitudinal data analysis (*10*, *24*–*26*).

Despite claims to the contrary, variability in average SWB age profiles has been consistently identified in the literature (*8*, *9*, *14*, *15*). For example, a recent meta-analysis of longitudinal studies from 29 countries showed slight increases in SWB from adolescence to age ~70, followed by a decline, resembling a wave-like or M-shaped pattern, instead of a U-shape (*10*). Assuming the existence of any single average age trajectory of well-being may detract from efforts to better characterize and explain individual trajectories of well-being across cultural and socioecological environments. In particular, under what conditions does well-being decrease in early adulthood and increase from ages 40 to 70, and what explains the more variable patterns from ages 70 onward?

One approach considers how variation in socioeconomic, cultural, and epidemiological conditions, operating at different levels (e.g., family and community), might be expected to lead to diverse age trajectories of well-being between and within countries (*27*). The rising post-midlife portion of the U-shaped well-being curve is often viewed as counterintuitive given strong links between health and SWB (*28*) and declining physical health after midlife. An uptick in well-being despite potentially declining health is usually explained by increasing appreciation and acceptance with age, especially after the strivings and competition of early and middle adulthood, combined with better emotional regulation, and adjustment of aspirations (*29*, *30*). While the generalizability of the U-shape remains controversial, the robustness of a midlife nadir is better supported empirically, including in studies that criticize the U-shape [see, e.g., (*31*)]. One possibility is that the midlife SWB nadir and post-midlife increase reflect something about modernity, more specific to high-income countries such as income security, pensions, and other social welfare programs (*32*). An increase in well-being from midlife to the 70s may be most evident in high-income countries if physical health does not decline appreciably, especially if employment in a largely urban information and service economy is compatible with modest health declines managed by affordable health care. However, U-shaped well-being age profiles have also been recently identified in several low-income countries (*6*, *21*). These samples might nonetheless reflect urban citizens with institutionalized retirement and some degree of social security. As recent overviews and meta-analyses attest, most studies of age profiles of SWB are confined to high-income countries, and broader sampling is needed, especially “…samples from non-Western countries and more diverse samples in terms of ethnicity” [(*10*), p. 434].

Here, we build on the few earlier studies of well-being in rural agrarian populations (*33*–*36*), many of which do not support a U-shaped well-being age profile, and instead show some evidence of lower SWB with age. We hypothesize that SWB trajectories with age are more variable than usually appreciated, especially when focusing on more rural, subsistence-oriented settings. In most rural subsistence populations (e.g., hunter-gatherers, horticulturalists, and pastoralists), physical activity is required for efficient food production and processing, mobility, and many other work tasks. In the absence of pensions and other means of financial assistance, care and well-being are tied to one’s social value within multigenerational support networks. Injuries, sickness, disability, frailty, and other forms of diminishing physical capacity associated with aging, especially with minimal healthcare, might reduce that value to others. If midlife slumps are nearly universal, then they should be apparent even in contexts without formal retirement, institutionalized social security, and where physical aging takes its toll. Alternatively, we hypothesize that in small-scale subsistence communities, well-being will not increase with age, especially during the period from ages 45 to 70. However, where older adults can augment their social value by other means, particularly through activities requiring less physical vigor (e.g., becoming skilled shamans, caretakers, leaders, advisors, storytellers, and community builders), their sense of self-worth and well-being may be buffered with age, by maintaining important (and respected) social roles. Where substitute roles relying less on physical vigor are minimal or lacking, we expect lower well-being with age. Given variation within and between populations in how people experience aging, we further propose that adjusting for indicators of physical health and productivity should account for much of the decline in well-being with age. To test our hypothesis, we assess age profiles of well-being using three well-suited datasets among rural, forest-dwelling populations living a large subsistence lifestyle. Study 1 involves SWB ratings among three Indigenous populations: Baka and Punan hunter-gatherers, and Tsimane’ horticulturalists (*37*). Study 2 consists of SWB measures and a richer set of covariates among rural forest users from 23 low-income countries (*38*). Study 3 is a longitudinal study of depressed affect in Tsimane’ horticulturalists. The longitudinal aspect of the third study provides an opportunity to evaluate SWB without some of the pitfalls of cross-sectional data. Our focus here on depression also provides a different lens for evaluating SWB. As Diener and colleagues (*39*) pointed out, cognitive appraisals of life satisfaction, the usual measure in happiness age trajectory studies, reflect only one aspect of SWB. Another important aspect includes affective components (positive and negative). Depressed affect, consisting of 16 items instead of just one, therefore reflects a more comprehensive and complementary measure of SWB.

Across the three studies, spanning several data collection and analytical methods, geographical, ecological, and cultural environments, we assess the relationship between SWB and age. We find only sporadic evidence for both a midlife slump and a U-shaped relationship between SWB and age. Age profiles are mixed and instead support a decline in SWB after middle age, mediated in part by diminished functional capacity and involvement in social networks. In Discussion, we address how our findings contribute to ongoing debates in the social sciences about SWB trajectories over the life course.

## RESULTS

### Study 1: Baka, Punan, and Tsimane’

**Fig. 1.**
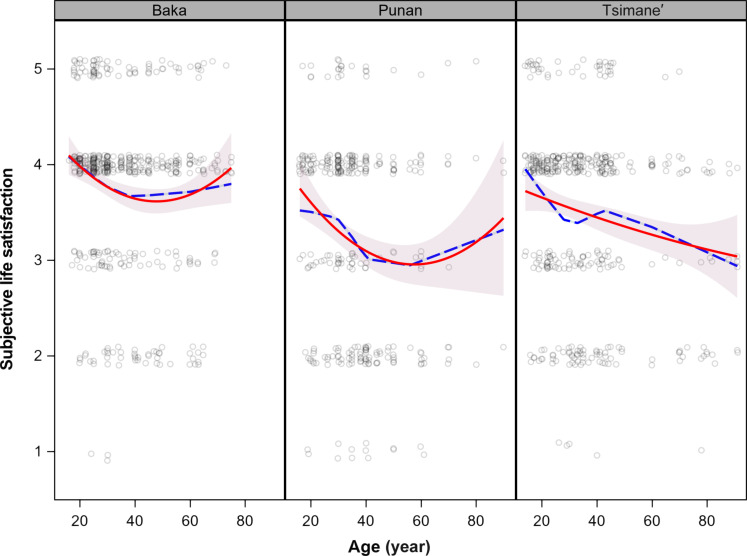
Subjective life satisfaction scores among Baka, Punan, and Tsimane’. Loess (dashed blue) and OLS regression (in red) using age and age^2^. The midlife trough is 47.5 years for Baka and 57.6 years for Punan.

Adults from three subsistence-oriented populations (*n* = 110 Punan, *n* = 223 Baka, and *n* = 135 Tsimane’) responded to a subjective life satisfaction scale as part of a larger study on the value of local environmental knowledge (sample characteristics are provided in table S1) (*40*). On a five-point scale, where 1 means “very bad” and 5 means “very good,” average subjective life satisfaction ranges from “fair” (3) to “good” (4) (3.2 in Punan, 3.4 in Tsimane’, and 3.8 in Baka). Overall, 63.4% of respondents said their lives were good or very good (4 or 5), and 20.2% said their lives were not good (1 or 2). Figure 1 shows second-order polynomial regression and loess fits of SWB with age. A positive quadratic age term (age^2^) and negative age term are consistent with a U-shape. A U-shape is found for Baka and Punan, albeit with high uncertainty at later ages, with the lowest SWB scores at age 47 years for Baka and 58 years for Punan. On the other hand, life satisfaction declines throughout adulthood among Tsimane’. The difference between average high and low spans between 0.4 and 0.8 units on the five-point scale. Women show lower life satisfaction than men among Punan and Tsimane’, but age profiles do not vary by sex. These quadratic age relationships are statistically significant for Baka and Punan, whereas only a linear decline is significant among Tsimane’ (table S2). When using ordered logistic regression instead of ordinary least squares (OLS) regression, we observe similar patterns of life satisfaction with age (see table S2).

Adjusting for sex, the full sample shows a U-shape, with a trough much later than typically reported in post-industrialized populations, at age 67.8 years. Including fixed effects for population and for effects of age and age^2^ to vary by population reveals patterns consistent with Fig. 1 (table S3). Modeling the probability of high or low life satisfaction also shows results consistent with Fig. 1 (see fig. S1). Using broad age categories and random intercepts for participants, SWB is significantly lower in middle adulthood among Baka and Punan but is not significantly higher at older ages (table S2). Using 5-year age categories, the age profiles become more erratic, especially for Tsimane’ (fig. S2), likely due to the small sample size and imprecise age estimation.

Adding sociodemographic confounders (years of schooling and household size) and mediators (wealth and income, and sickness) does not substantially alter the magnitudes of the global and population-specific age profiles but does reduce the statistical significance of the age terms (compare tables S2 and S3 with table S4). More days spent sick enough to disrupt work activities had the strongest and most consistent effect on lowering SWB.

### Study 2: Forest users in 23 low-income countries (PEN)

**Fig. 2.**
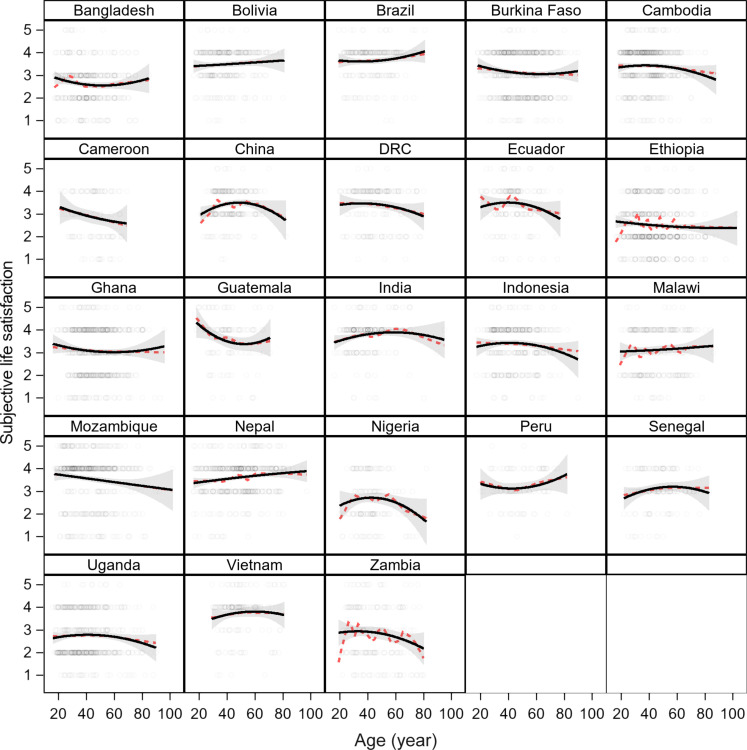
Subjective life satisfaction in PEN sample of forest-dependent sites in 23 countries (study 2). Loess smooth (in dashed red) and second-order OLS regression (black) by age.

From rural forested regions of 23 low-income countries, 6970 adults were sampled to complete a subjective life satisfaction scale as part of a larger study (sample descriptives provided in table S5) (*41*). On a five-point scale (from 1 to 5), subjective life satisfaction ranges from an average low of 2.3 in Ethiopia to an average high of 3.9 in India (fig. S3). Overall, 48.9% of respondents said their lives were good (4 or 5), and 29.0% said their lives were not good (1 or 2). Figure 2 models life satisfaction scores using a loess smooth and OLS regression containing age and a quadratic age term (see fig. S4 for disaggregation by sex). A U-shape is apparent in 8 of the 23 country sites (means ± SD nadir: 67 ± 31 years; range: 35 to 103 years), while an inverted U—consistent with peak midlife contentment—is observed in 14 countries (means ± SD peak: 55 ± 36 years; range: 32 to 167 years). Of the eight U-shapes, two of them reflect decline over most of adulthood (sites in Cameroon, Ethiopia), and two show troughs after age 59. The remaining sites show no change (sites in Bolivia and Malawi) or a slight decline with age (Mozambique site). In any case, site-specific regressions show that few of these age relationships are statistically significant at conventional levels (table S6). Using ordered logistic regression based on a reduced three-point scale (1 to 2 = low, 3 = average, 4 to 5 = high) instead of OLS changes only a few results from the OLS (e.g., sites in Cameroon, Mozambique; table S7: model 2 versus model 3). Overall, the percentage of variation explained by age across populations is very low (<5%). We also consider the probability of reporting higher life satisfaction (4 or 5) in a logistic regression that includes age and age^2^. Results are qualitatively similar to those from the OLS and ordered logistic regressions (figs. S5 and S6), and a few of these relationships are statistically significant (table S2). Last, we examined observer ratings of smiling and laughing to compare with SWB. Figure S7 shows that 17 of the 23 countries have similar profiles of positive affect with age as the life satisfaction age profiles of Fig. 2.

**Fig. 3.**
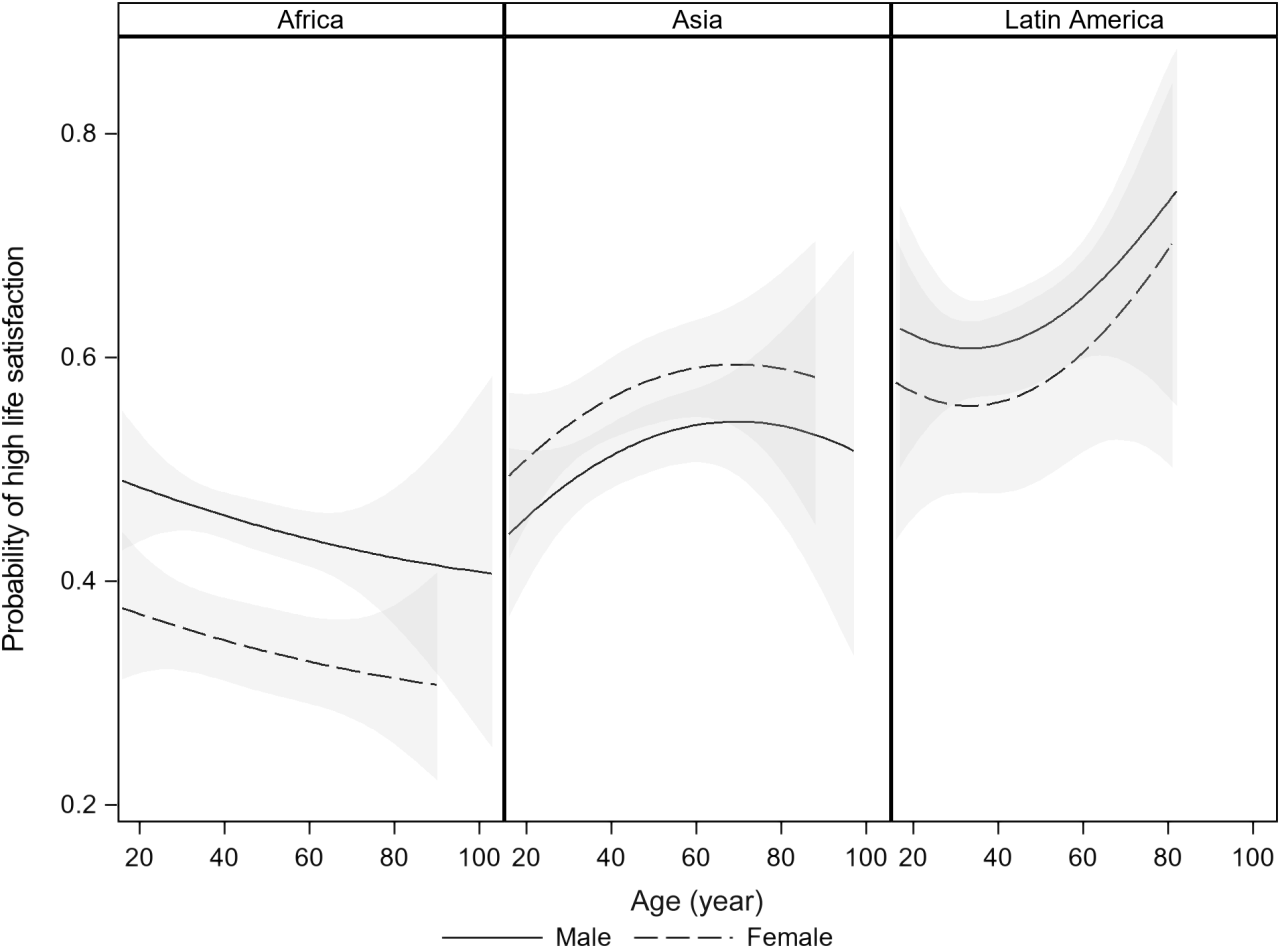
Probability of high life satisfaction in forest-dependent communities across continents (study 2). Random intercepts are used for country-specific sites. None of the age effects are significant at conventional levels.

Pooling at the continent level to increase sample size, Latin America shows a U-shape, consistent with the site patterns in Brazil and Peru, but with a nadir at age 44 years. Asia shows an inverted U with a peak at age 56 years, and Africa shows a decline in both raw scores and probability of high satisfaction over adulthood (Fig. 3 and fig. S8). Analyzing age profiles by continent does not alter the significance of the age terms, neither for OLS nor for ordered logistic regression, though it shifts Latin America’s nadir to 26 years (tables S6 and S7). Age is only significant in Africa when modeled with a linear term, showing a decline (OLS: *b* = −0.004, *P* < 0.001; ordered logistic: *b* = −0.007, *P* = 0.001). Adjusting for a large set of variables on sociodemographic characteristics and experiences of adversity does not qualitatively alter the age effects, although the U-shape in Latin America becomes marginally significant in the OLS model, with a trough at age 49 years.

Next, we perform OLS and ordered logistic regressions of the global sample, including random effects for sites nested within continents. Globally, there is an inverted U with a slight peak at 16 years, but not statistically significant (table S8: model 2). Only a slight linear decline with age is significant (table S8: model 1). Adding sociodemographic variables and indicators of recent adversity does not qualitatively change results (table S8: model 3). While age effects are minimal, these other variables are associated with life satisfaction in the expected direction. Individuals who are married, wealthier, educated, and living in larger households report higher SWB. Those who experience recent illness or death in their families, major crop or livestock loss, or other shocks report lower SWB compared to those who did not experience any of these. Those who say they have others to rely on for help and can trust other community members are more satisfied than those who cannot. These significant effects are generally of a larger magnitude than the age terms (standardized regression estimates of 0.03 to 0.12 versus 0.04 to 0.05 for age terms) and are larger than the sex difference (0.02), which drops out in the full models.

**Fig. 4.**
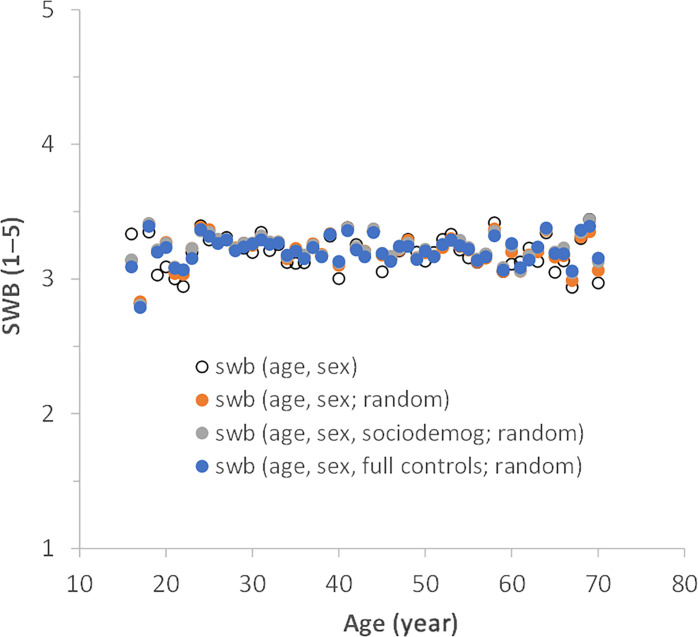
Subjective life satisfaction in forest-dependent communities from 23 countries (study 2). Age is treated as a categorical variable in OLS regressions. Four sets of model predictions are shown: (i) age and sex (open circles); (ii) age, sex, and country as a random effect nested within continent (red circles); (iii) age, sex, sociodemographic controls, and similar random effects (gray circles); and (iv) age, sex, full set of controls, and random effects (blue circles). Covariates evaluated at sample means.

As a final robusticity check, we consider age as a categorical variable so as not to assume any functional form for the relationship between life satisfaction and age (Fig. 4). We limit the sample to include only participants aged 70 and younger (*n* = 6641) because the increase from a midlife slump is reported to be most common up through age 70. Figure 4 shows that life satisfaction increases in the late teenage years to the ages of 20 to 25 years, and then remains relatively flat thereafter. This pattern is not altered by including a large set of controls nor by accounting for the nonrandom geographic structuring of the data (individuals from countries nested within continents). Grouping ages into 5-year age categories because of potential age uncertainty does not change results.

### Study 3: Tsimane’

#### Cross-sectional

**Fig. 5.**
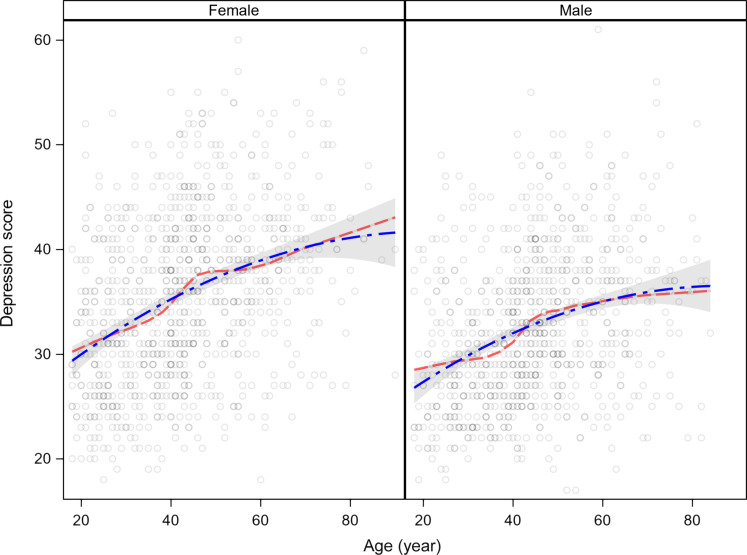
Depressed affect score by sex for Tsimane’. Trendlines are from loess and OLS regression including age (*b* = 0.370, *P* < 0.0001) and age^2^ (*b* = −0.002, *P* = 0.002), *R*^2^ = 0.16.

A sample of 1872 Tsimane’ were interviewed about symptoms of depression using a 16-item inventory (for sample characteristics, see table S9), ranging from 18 to 60. Means ± SD depressed affect scores are 35.5 ± 8.1 for women and 32.6 ± 7.3 for men. Figure 5 shows depressed affect scores for Tsimane’ women and men using second-order polynomial and loess regression. Although the quadratic age term is significant and negative, depression score rises over much of adulthood and increases only modestly after age 70. Restricting ages to 70 years increases the magnitude of the quadratic age term and reduces the slope, which is still significant (fig. S9). Similarly, the probability of having a top-quartile depression score is greater with adult age, particularly among women (fig. S10). Considering age as a categorical variable shows a similar increase in depression scores over adulthood (fig. S11). Interviewer ratings of positive affect also do not show a U-shaped relationship with age but instead an inverted U (fig. S12).

**Fig. 6.**
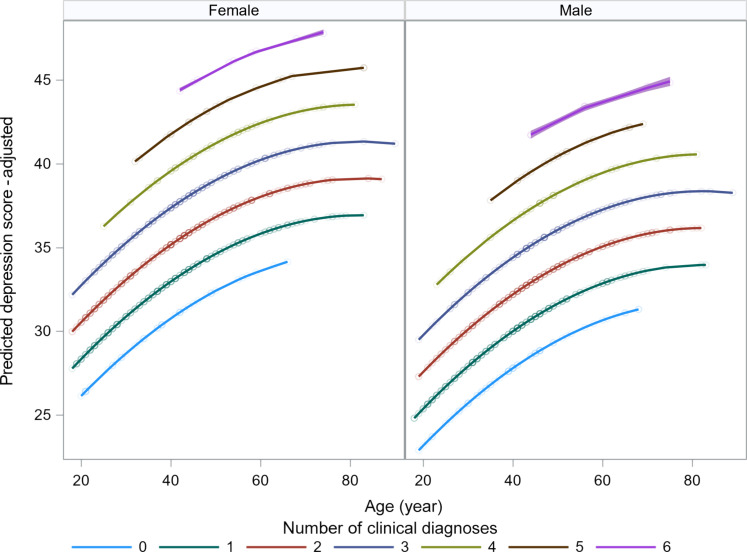
Tsimane’ depression score and medical diagnoses. Diagnoses reflect clinical condition categories evaluated by Tsimane Health and Life History Project physicians during routine checkups.

Including the number of physician-diagnosed health problems in regression models helps explain additional variance in depression score but does not substantially alter the age profiles shown in Fig. 5 and fig. S11 [age: *b* = 0.370 versus *b* = 0.360 (adjusted for diagnoses); age^2^: *b* = −0.0020 versus *b* = −0.0022; all *P* < 0.001]. The magnitude of health effects is strong (table S10: models 2 and 3). For example, Fig. 6 shows that physician-diagnosed health status spans a broader range in depression scores than does age. Age alone explains 11% of the variation in depression scores, whereas the number of medical diagnoses explains 16%.

We next restrict the age range to 40 to 70 years, where SWB has been hypothesized to increase from its nadir in many populations, and where we have additional health-related variables to account for functional capacity from the Tsimane’ Health and Life History Project (see Materials and Methods) (*42*). In models that also consider self-reports of musculoskeletal pain, problems performing productive activities, activities of daily living (ADLs), problems seeing, and beliefs about one’s own health, the age effect is reduced markedly, whether considering only a linear age term or age and age^2^. For example, in the full model, the positive effect of age on depression score is reduced by 42% (*b* = 0.117, *P* < 0.0001 in the baseline model with sex versus *b* = 0.068, *P* = 0.048 in the fully adjusted model). From age 40+, the total variance explained by the health variables alone, adjusted for the number of variables, is 26%, versus 3% by age alone, and 4% by age and age^2^ alone. The number of medical diagnoses alone explains 12% of the variance in depression scores. As an independent check, we assessed the age profile of interviewer ratings of happy affect, with a score ranging from 1 (did not smile or laugh once ~10 min before and after the interview) to 4 (smiled and laughed often). OLS and loess fits show a significant inverted U-shaped happy affect curve with age (fig. S12), with a peak at 60 years (56 years in women, 64 years in men).

#### Longitudinal

The means ± SD time between repeated depressed affect evaluations of the same people was 2.9 ± 1.8 years. Overall, depression scores within individuals are only weakly correlated (Pearson *r* = 0.092, *P* = 0.008), and when comparing within-individual scores by age group, they are only significantly correlated among adults aged 40 to 59 (*r* = 0.106, *P* = 0.014), consistent with the notion that Tsimane’ depressed affect is more acute than chronic. We model depression score as a function of age and other covariates, clustering standard errors and using fixed effects for individuals, covering 1804 observations among 686 Tsimane’ adults aged 18 to 92. In a baseline model without fixed effects, depression score increases slightly over adulthood (table S11: model 1). Once fixed effects are added, the relationship is more of an inverted U with a late peak at age 54 years, though the relationship is not statistically significant (table S11: model 2). Adding health-related covariates does not qualitatively change these results (table S11: models 3 and 4).

A second method for assessing the age profile is a graphical approach, based on the first derivative notion that the rate of change in depression should be negative with respect to age if depression is an inverted U with age (i.e., consistent with happiness being U-shaped), with the age of nadir being the crossing point of the *x* axis. Likewise, the rate of change would be positive with age if depression is instead U-shaped, and no different from zero if there is no relationship between depression and age (*15*). Our graphical results show a slightly negative, but statistically insignificant, slope that crosses the *x* axis around age 27 years (fig. S13). Fuller models do not substantially change the slope (table S11: models 3 and 4).

## DISCUSSION

Despite widespread recognition that there is no single age trajectory of human happiness, the notion that the average level of happiness takes a convex turn across the life course is still touted by U-shape proponents to be a “completely universal human phenomenon” and a “first-order discovery about human beings that will outlive us by hundreds of years” (*7*). However, to date, much of the evidence in support of the U-shape comes from cross-sectional studies of large, urban samples from high-income countries. Here, we explored age profiles of subjective life satisfaction in 27 samples, representing more than 9000 rural, largely subsistence-oriented participants from Latin America, Africa, and Asia. We used similar methods as those prevalent in the literature reporting U-shapes, and in two studies (2 and 3), we used two different measures of SWB. In addition, in one population (Tsimane’, study 3), we also present longitudinal data and more detailed affective and cognitive self-reported symptoms of lower well-being. Where possible, we addressed the four common statistical pitfalls that typically bias SWB regressions: (i) quadratic specification bias, which results from relying only on a quadratic age term; (ii) omitted variable bias, which results from not including confounds; (iii) overcontrol bias, resulting from including mediators of happiness; and (iv) mortality selection bias, which results from relying only on cross-sectional studies (*43*). While we uncovered some evidence of the U-shape in several samples (e.g., Punan in study 1; sites in Peru, Brazil, in study 2), the nadir in these samples was not consistently in the 40- to 50-year age range that is typically reported in studies conducted in high-income countries [see, e.g., (*44*)]. We found many cases of no significant changes in SWB throughout adult life or of inverted U-shapes more consistent with midlife peaks than crises. The latter is particularly noteworthy because at least one review of the field concluded that the notion of an inverted U-shape could be dismissed on both empirical and theoretical grounds (*9*).

In the face of health decline and social losses, the rise in well-being from the midlife slump observed mostly in high-income countries has been called a paradox. Our results suggest that there may be no paradox, at least among rural non-industrialized populations with low access to formal institutions providing social security and other benefits. Our findings suggest that well-being often tends to be lower, or it remains relatively unchanged, from ages 45 to 70 years.

Like many other studies [see, e.g., (*39*, *45*–*48*)], we also find that socioeconomic, health, and other time-varying factors affect SWB. In study 2, we found that income, education, household size, and marriage were all important SWB predictors. Shocks like crop or animal loss, illness, and death lower SWB. Other factors, like perceptions of a trusting and generous social environment, were associated with greater SWB. Similarly, health problems, trouble performing daily productive tasks, mobility limitations, and negative attitudes about future health all predicted lower SWB among Tsimane’ (study 3) and, in combination, accounted for much of the depression reported among older adults. In rural subsistence contexts, these mediators of SWB are not always tightly linked to age. For example, Pearson correlations of health and functional capacity mediators ranged from 0.10 to 0.31 among Tsimane’. In general, those factors combined eliminated any small age effects in simple models. These findings support the notion that age effects are often confounded by life-altering factors that vary across the life course and by sociocultural context.

The few other studies of subjective life satisfaction in small-scale societies lend additional support to our findings. Project AGE, conducted using standardized interviews in five countries in the 1980s, showed that well-being among Ju/’hoansi (!Kung) hunter-gatherers and Herero pastoralists was highest at ages 30 to 59 and lowest among those aged 60+ years (*36*); Ju/’hoansi data support an inverted U, while well-being among Herero was lower with age (fig. S14). A comparison of Hadza hunter-gatherers with Polish citizens also showed no significant age-related differences among Hadza adults, but lower well-being with age throughout adulthood in Poland (see fig. S15) (*35*). There was also no significant U-shape among Iñupiat and Yup’ik of Arctic Alaska (*n* = 530), which was instead mostly influenced by health status, family ties, and social support (*49*). Affect balance (a measure of having more positive than negative emotions) was reported to vary inversely with age among a small sample (*n* = 127) of Kenyan Maasai pastoralists (*50*). Last, a study of well-being using three measures (life satisfaction, affect balance, and momentary affect) in coastal communities of the Solomon Islands and Bangladesh found no differences in well-being between adults aged <50 years and aged 50+ (*51*).

Two other studies suggest that the patterns we report are not limited to Indigenous peoples or forest-dependent market-limited societies but may be common to rural or low-income settings—a context poorly represented in the SWB literature (*9*, *10*). A panel study of life satisfaction, depression, and anxiety among adults age 45+ in rural Malawi (*34*) showed that well-being in all three measures decreased with age throughout adulthood. Cohort differences did not explain the age effects, and half of the age effects were mediated by declines in physical health and disability. Rural Malawians also report more chronic pain with age, especially after age 45 (*52*), as do the Tsimane’, consistent with study 3 (*53*). In a review of depression prevalence studies in low- and middle-income countries using representative country-level data, Banerjee and colleagues (*54*) report age-related increases in depression in China, India, Mexico, South Africa, and Malawi (based on the same study mentioned above) (see fig. S16). They conclude that contrary to the common expectation of rising well-being with age, there is instead an “unseen epidemic of poor mental health among the elderly in developing countries.”

This study not only contributes to the growing body of evidence against the notion of a universal U-shaped happiness curve with age but also suggests that midlife lows may be less commonly represented in low-income countries, especially in rural, non-industrialized contexts. Cluster analyses of SWB across 81 countries similarly show that age-related declines in well-being appear throughout low-income countries in Africa and South Asia, while a decline with age followed by a stable flat level after reaching the nadir is reported for the United States and Australia. The U-shape is observed mostly in Europe and middle-higher income areas of South America (*27*). Age-related declines in well-being have been consistently reported in economically depressed regions of Eastern Europe (*23*, *35*, *55*), and the declines in SWB with age that we report here throughout Africa are consistent with other studies (*34*, *54*). Common characteristics of countries in the sustained age-related decline cluster include lower educational attainment, higher gender inequality, and lower human development index. Such age-related declines may be more common than previously thought, even in European countries, using more robust fixed effects models in longitudinal datasets (*43*).

To date, most advocates of the U-shape have been economists, whereas many psychologists have either argued against or trivialized the importance of the U-shape even in the Global North (*8*, *9*, *56*). A recent large-scale study by psychologists using Gallup data from 1.7 million people from 166 countries showed mostly declines (albeit U-shaped), or no changes, in both life satisfaction and positive affect from ages 20 to 80 years (*46*). Declines, when observed, were similar in magnitude to being unmarried or unemployed, but smaller than feeling that your life has no purpose or meaning, or that you have nothing to give to others.

Together, it appears that there is no clear midlife SWB dip under more adverse conditions. Instead, well-being either remains relatively flat or worsens with age. What conditions might foster the maintenance of, or even an upturn in, well-being after middle adulthood in the face of adversity? Current explanations for a rise in SWB from ages 50 to 70 years invoke shifts in aspirations, perspectives, and ambitions that presumably lead to more contentment despite health declines and social losses (*13*, *30*, *57*). The upturn should be most visible where satisfaction in financial, social, and other life domains remains high. These domains make up a large part of overall well-being in predictable ways, contrary to strict notions of hedonic adaptation (*58*). A stability-despite-losses pattern may be expected in high-income countries where health declines do not necessarily erode economic and social security. Consistent with this notion, a meta-analysis showed that health was more highly correlated with SWB in low-income countries than in high-income countries (*59*). In rural, largely subsistence-oriented contexts, especially where health is likely more compromised, it may be difficult to maintain livelihoods and security. Even when these are maintained through kin and informal networks, dependency and perceived burden on others may hinder well-being. In the ethnographic record, sustained participation in valued activities is associated with elders earning respect (*60*). Likewise, elder neglect, abandonment, and other forms of “death-hastening” behavior are more common when elders are physically impaired, and where livelihoods may be hard to maintain (*61*).

Even in high-income countries where U-shapes have been identified, elements of this logic may play out. European countries with lower per capita GDP are more likely to show fluctuating or age-related declines in SWB (*44*). The convexity of the U-shape is weaker during periods of economic recession (*62*). Low-income subgroups are also more likely to show age-related declines in well-being (*63*). When livelihoods are difficult to maintain through later years, especially where welfare programs are minimal, steeper age-related declines in well-being have been reported (*23*). This is consistent with psychological evidence in post-industrialized countries where prolonged and unavoidable stresses compromise any age-related advantages in social and emotional functioning in later adulthood (*57*, *64*). Beyond the mid-70s, adjustment and coping may be more commonly strained in both low-middle and high-income contexts in the face of health-related and social factors, accounting for the late-life declines in well-being often reported even where there is a U-shape up through age 70.

Another possibility worth exploring focuses on the middle part of the age curve. Is there something particularly stressful about midlife in places where U-shapes are found? The combination of childcare, raising teenagers or young adults, alongside occupational obligations in the more nuclear family-oriented urban settings may lead to lower well-being in midlife. Across 29 European countries, happier countries show earlier turning points in midlife by over a decade in comparison to less happy countries (*44*). Furthermore, U-shapes, even when observed, are flatter among subgroups with strong social relationships at home, work, and in the community (*65*).

One important limitation of cross-sectional data occurs if individuals of different ages vary not only because of aging but also due to differences in cohort experiences and expectations (i.e., affecting happiness “set points”). For example, although well-being appears to have an inverted U, or decline, with age in two US samples, this age pattern reverses after taking into account that earlier cohorts had lower baseline well-being, especially those born during the Great Depression era (*66*). While we cannot address cohort effects in studies 1 and 2, several points are worth mentioning. First, the Malawi study cited above showed that considering cohort effects did not alter age profiles of well-being. Second, the majority of Tsimane’ older adults (study 3, 73.8%, *n* = 397 adults age 55+), despite showing higher depression scores, claim in an ongoing survey that the lives of older adults are actually better now than they were in the distant past (table S12). Tsimane’ elders today would thus be expected to have higher well-being than those elders living in the past. Third, the large meta-analysis of longitudinal studies mentioned earlier found no effects of the cohort on the shape of the lifespan trajectory of SWB (*10*). Last, given that most U-shape patterns have been identified in cross-sectional studies, our set of mostly cross-sectional samples should have been more poised to detect them.

Other limitations often mentioned when U-shaped patterns are not found include small sample sizes or unrepresentative samples. Nation-level representative samples are usually >5000 participants, a tough bar to reach in small-scale societies. There are only 1300 Hadza, so a sample size of 145 is small in absolute terms, and with respect to statistical power, but not unreasonable relative to the adult population size. The Tsimane’ sample from study 3 is relatively large, including 1872 of the 6800 adults age 20+ in the population. Nonparticipation in these studies, if anything, should be of those with lower well-being, not higher. The individual country-specific samples in the PEN study were small (*n*’s ranging from 86 in Peru to 570 in Ghana), but aggregating at higher levels, like continent (*n*’s from 871 to 3752), and to the entire PEN sample (*n* = 6987) showed similar patterns largely inconsistent with a U-shape. If age effects are strong enough and large enough in magnitude to be meaningful, then it should not require thousands of participants to detect significant age effects. It should also be noted that the PEN study was heavily (75%) male-biased (table S5), and with samples from three countries >90% male. In addition, country-level datasets, though large, are also mixes of people with diverse backgrounds. Net age profiles from such mixed samples may not apply to all subgroups and may not be very informative. The samples presented here are more culturally and socioeconomically homogeneous. We view this as a study strength.

Another limitation often cited in cross-sectional studies is the potential for mortality selection and attrition bias to give a spurious post-slump upward rise in well-being. Our samples come from higher mortality settings, which should bias well-being to rise even more steeply with age. If participants are happier on average than nonsurvivors and nonparticipants, then the bias implies that that our post-50 curves should be even lower than they actually are. Selection bias therefore seems an unlikely explanation for the patterns we report here.

A final limitation is that SWB manifests in more ways than the cognitive evaluation of life satisfaction or self-reported happiness (*67*). While study 3 and others cited here involve aspects of positive and negative affect, we did not have multiple types of well-being measures for every study, and age profiles sometimes vary by measure. Studies also do not address eudaimonic aspects that tap into life’s meaning and purpose.

To conclude, understanding the age trajectory of happiness is a contentious enterprise marked by strong claims, post hoc theorizing, and methodological mayhem (*8*, *23*, *43*, *46*). While methodological inconsistencies are responsible for some of the mixed empirical findings in the literature, clarity on the larger question about how and why happiness changes with age (and its close cousin: how happiness varies among people of different ages) is unlikely to come from only improving and standardizing statistical methods. To date, non-industrialized populations have been only sparsely sampled, including when low-income countries are included in studies. Here, we showed that rural, largely subsistence populations show diverse average age trajectories of happiness, with well-being more likely to be lower, not higher, after midlife. Our findings lend further support to the idea that the U-shape happiness age trajectory does not reflect something fundamental about people. The variance explained by these average age trajectories is consistently small, suggesting that a better understanding of individual life trajectories and their determinants will be more fruitful. The notion of variable trajectories fits within the strength and vulnerability integration framework of S. Charles (*68*), wherein the net effect of the advantages of aging (e.g., positivity bias and emotional regulation) weighed against its harms (e.g., physical frailty, social losses, and isolation) should determine the shape of the SWB trajectory in later adulthood.

Together with the prior studies linking adversity to lower SWB mentioned above, our findings among small-scale forest users are also consistent with the evolutionary hypothesis that dissatisfaction, especially when expressed as low mood and rumination, may function to help individuals analyze and resolve complex problems without distraction (*69*, *70*)—problems that may arise as a consequence of lower productivity and perceived social value, social losses, and higher morbidity with age. Depressed affect among Tsimane is more common among those reporting having more problems (Spearman *r* = 0.56, *P* < 0.0001; adjusting for age and sex), including recent death (*r* = 0.45) a lack of social support (*r* = 0.44), social conflicts with close kin (*r* = 0.43), and food insecurity (*r* = 0.30). Low mood may also serve as a credible signal of need, to elicit support from others (*71*) and avoid social exclusion (*72*). Whether depressed affect helps individuals devise novel strategies for improving their circumstances warrants further consideration.

Well-being is an important element of healthy aging, and governments are increasingly taking stock of national SWB as a desirable form of wealth (*73*). By 2030, the global adult population ages 50 years and older is expected to reach 2.3 billion, and unipolar depression is projected to be the largest single contributor to global morbidity (*74*). A better theoretical and empirical understanding of the determinants of adult well-being trajectories is needed to help ensure successful aging everywhere, and not just in the West.

## MATERIALS AND METHODS

### Study 1: Baka, Punan, and Tsimane’

As part of a cross-cultural project on the adaptive value of local ecological knowledge, SWB was studied among two foraging populations, the Baka of the Congo Basin and the Punan of Borneo, and Tsimane’ horticulturalists, between 2012 and 2013 [see (*37*) for details]. SWB was queried: “Taking everything into consideration, would you say your life is 0 = very bad, 1 = not good, 2 = fair, 3 = good, 4 = very good,” followed by verbal explanations for their answers. Overall, roughly half of people in each of the three groups said life was “good,” with more reports of “very good” than “very bad.” Individuals were sampled every 3 months, up to four times. In total, 474 individuals were sampled 1174 times: Baka (*n* = 223 inds/460 ob, mean age = 36.4 ± 15.0 years), Punan (*n* = 110/309, mean age = 36.6 ± 14.5 years), and Tsimane’ (*n* = 135/405, mean age = 36.3 ± 18.1 years). Half (49.5%) of participants showed consistency across quarters, and a quarter (25.1%) differed by one level. The few with larger differences stemmed from life events like widowhood or birth (*37*). Poor health was given as the main reason for low well-being, especially among Tsimane’ and Punan. Additional variables available include the highest level of schooling, household size, household wealth, total annual income, and the maximum number of days unable to work due to sickness in the past 2 weeks. Informed consent was obtained from tribal governments and all study participants. All protocols were approved by the ethics committee of the Autonomous University of Barcelona (CEEAH-04102010).

### Study 1: Analysis

We use several analytic techniques common in the happiness literature to ensure robustness and avoid common biases. The first method is OLS regression of well-being using age and a quadratic age term. A positive age^2^ term and a negative age term are consistent with the U-shape. To avoid constraints imposed by a quadratic functional form, a second approach presents loess smooths and uses age dummies in OLS regression. The third is an ordered logistic regression on a reduced version of the subjective life satisfaction, with responses 0 to 1 lumped as “poor” and 3 to 4 lumped as “good.” Given sample size limitations, we also analyze life satisfaction using four age categories (<30, 30 to 44, 45 to 59, and 60+). In all models, we use a hierarchical approach, starting with a baseline model with age and age-squared (centered on mean to reduce collinearity), then adding sociodemographic confounders. Last, we add proxies of health or disability, where available, as potential mediators of well-being. Given repeat quarterly sampling, we include random intercepts for participant ID.

### Study 2: Rural forest users in 23 countries (PEN)

The CIFOR Poverty Environment Network (PEN) collected individual and household-level data from rural-living people in 23 low-income countries in Asia, Africa, and Latin America between 2005 and 2010 (*38*, *41*). Roughly 8000 households were randomly sampled from village censuses; villages were selected on the basis of stratification criteria according to land tenure regime, market involvement, vegetation type, and ethnicity. The first household head that was available was selected to be interviewed. The PEN study therefore had a strong female bias in sampling due to male absenteeism, with men constituting 75% of the total sample (see table S4). SWB was measured on a five-point scale in response to the question: “All things considered together, how satisfied are you with your life over the past 12 months?” Interviewer ratings of positive affect were assigned on the basis of observations of smiling and laughter, on a four-point scale (1 = minimal,…, 4 = pervasive). Control variables include household size, marital status, education level and income (absolute and relative), and recent household shocks, like job loss, income loss, and death in the household. The final sample of SWB with covariates is 6970 households. Before data collection, researchers obtained prior informed consent from the local authorities at village and regional levels, as well as from each household and individual participating in the research.

### Study 2: Analysis

As with study 1, we use OLS, logistic, and ordered logit models by population and aggregated by continent, as well as for the combined sample. We also use age as a categorical variable on the full sample, adjusting for the study population.

### Study 3: Tsimane’ depressed affect

The Tsimane’ Health and Life History Project has collected data on depressed affect (yoquedye’) from 2006 to 2015 using a 16-item inventory adapted from the Hamilton Depression Rating Scale and Center for Epidemiologic Studies Depression Scale [see (*33*) for details]. Symptoms queried include sadness, negative thoughts, lack of concentration, and persistent thoughts, among others. Each symptom was evaluated in reference to the past month on a four-point scale, from rare to always. Item scores were summed to generate an overall depressed affect score. Depressed affect was also assessed as having a score in the top quartile (>39). A total of 2774 depression scores were collected on 1872 individuals aged 18+ years (27% sampled twice, 9% sampled more than three times; mean time gap between observations is 2.9 ± 1.8 years). Interviewer observations of smiling were also recorded for most cases. Informed consent was obtained by Indigenous governing bodies, village leaders, and all study participants. Ethical approval was obtained from the Institutional Review Board of University of California Santa Barbara (#15-133) and Universidad Mayor San Simon, Cochabamba Bolivia.

### Study 3: Analysis

A previous cross-sectional study showed that Tsimane’ depressed affect score was lower at older ages and that the age effect was mediated by indicators of disability and productivity (*33*). Here, we first analyze a larger cross section of depression (both raw score and a binary indicator using the top quartile) as a function of age, age^2^, and sex. We adjust for several health indicators to assess whether physical function mediates age effects: a number of diagnoses assigned by project physicians, ADLs (problems bathing, eating, and dressing), self-reports of physical pain, problems performing daily economic activities (yes/no), and vision problems, as well as a composite health belief score that incorporates self-reported current health and future health (higher values connote worse health). While medical diagnoses exist for the full sample of adults, the other health-related measures are restricted to ages 40+ or 50+. As a robustness check, we assess interviewer ratings of affect, based on smiles and laughter (Pearson *r* = 0.21 between depressed affect score and interviewer affect rating). Last, we model longitudinal data in two ways: (i) we use both OLS and logit regression on the subset with 2+ observations to model the depressed affect at time *t*, as a function of age, age^2^, sex, and the additional covariates, clustering standard errors and treating individuals as fixed effects. We use fixed effects for individuals instead of random effects, given best practices by (*43*); (ii) Following (*15*), we model the annual change in depression score as a function of age. A negative slope passing through the origin at the midlife nadir is consistent with an inverted U depression curve, whereas a U-shaped depression curve should lead to a positive slope, and a flat relationship with age should be reflected in a slope not different from zero.

## Notes

**Correction (16 September 2025):** The original version of the article stated that ape caretakers in ref. 12 were from “Western countries.” This has been corrected to “high-income countries,” as not all the caretakers were from Western countries. The PDF and XML have been updated.
