# Supplementary material for: Subjective well-being across the life course among non-industrialized populations
Source: Sci Adv. 2024 Oct 23;10(43):eado0952. doi: 10.1126/sciadv.ado0952 (PMC11498220; doi:10.1126/sciadv.ado0952)
Supplement: Supplementary file 1 — Figs. S1 to S16 Tables S1 to S12 [file sciadv.ado0952_sm.pdf]

Science Advances
AAAS

# Supplementary Materials for

## Subjective well-being across the life course among non-industrialized populations

Michael Gurven *et al.*

Corresponding author: Michael Gurven, gurven@anth.ucsb.edu

*Sci. Adv.* **10**, eado0952 (2024)
DOI: 10.1126/sciadv.ado0952

**This PDF file includes:**

Figs. S1 to S16
Tables S1 to S12

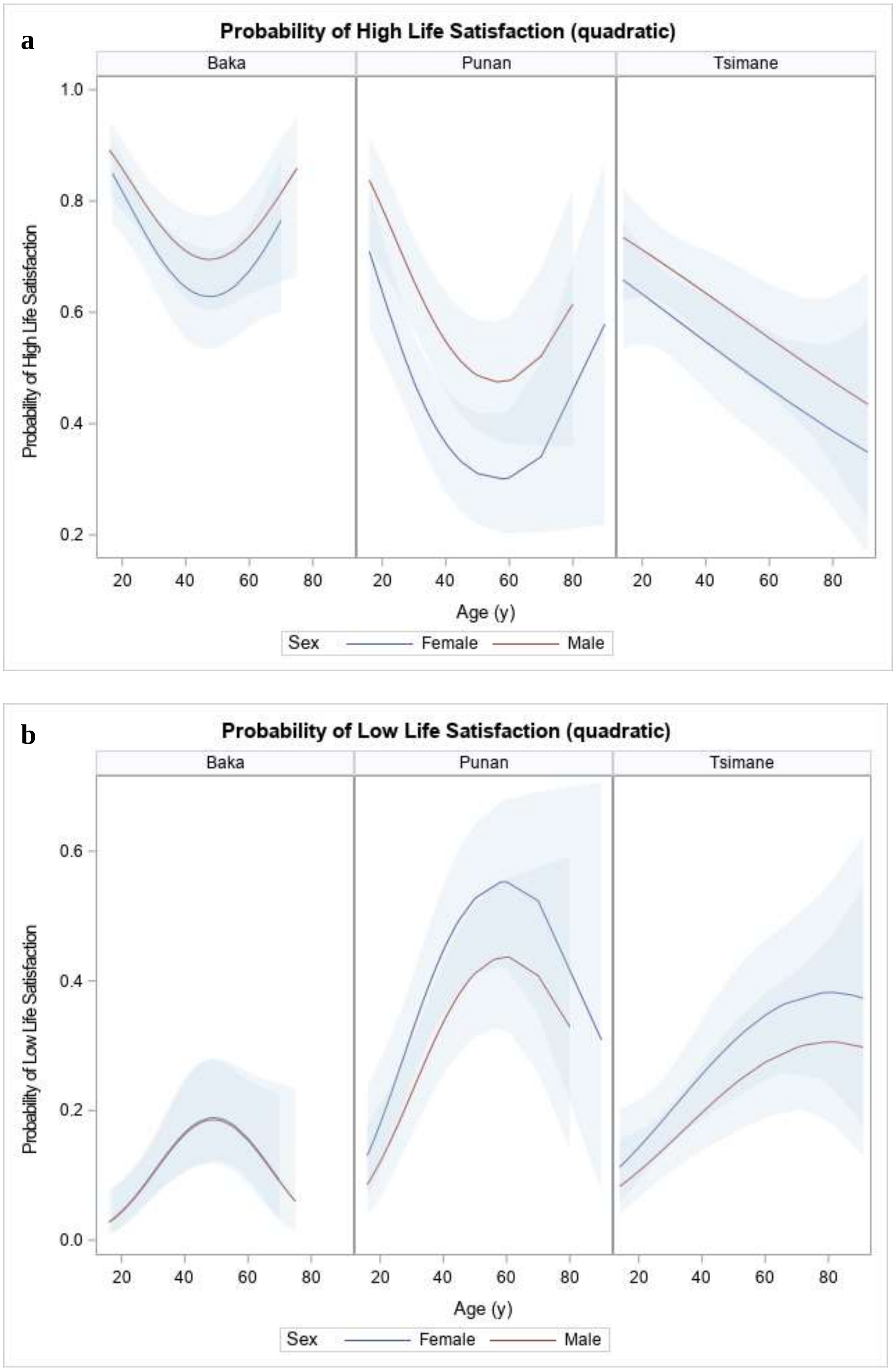


**Figure S1. Logistic regression of (a) High or (b) Low Life Satisfaction: Baka, Punan and Tsimane'.** Models include age, age$^2$ and sex.

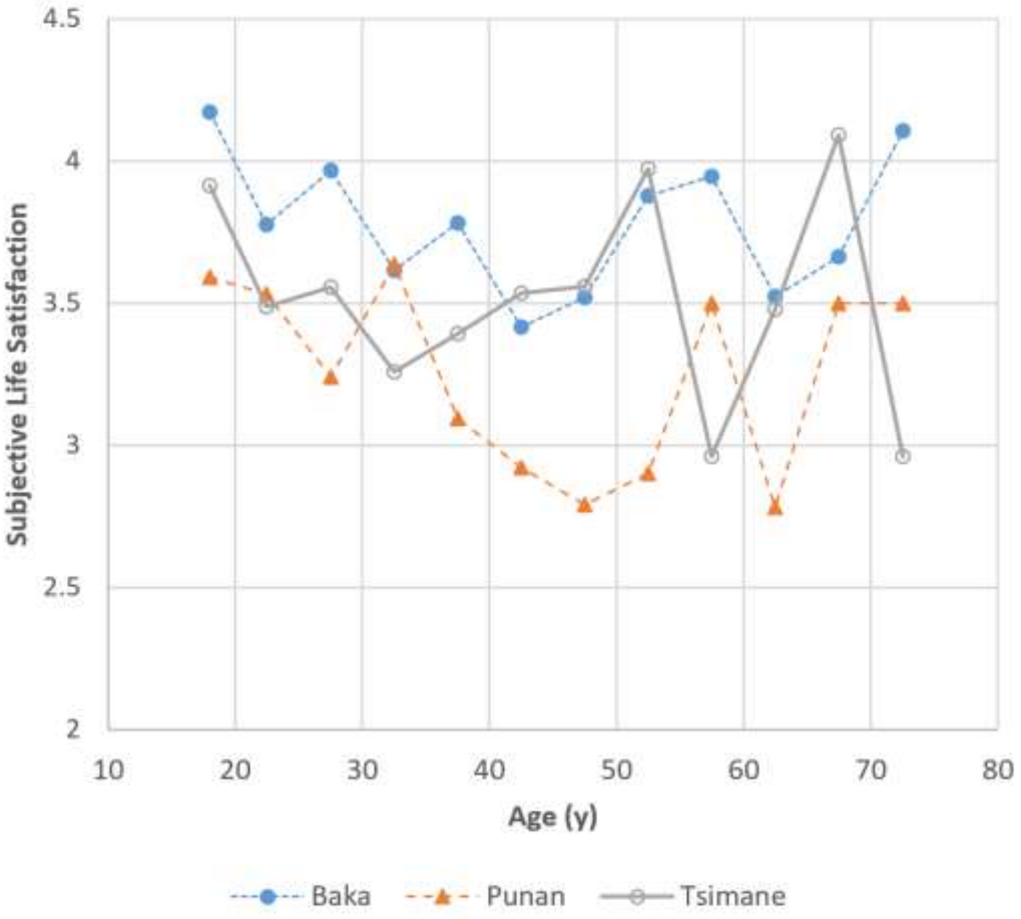


**Figure S2. OLS Regression of Subjective Life Satisfaction.** Age is 5-yr Categorical Variable. Adjusted for sex, and using individual ID as random intercept.

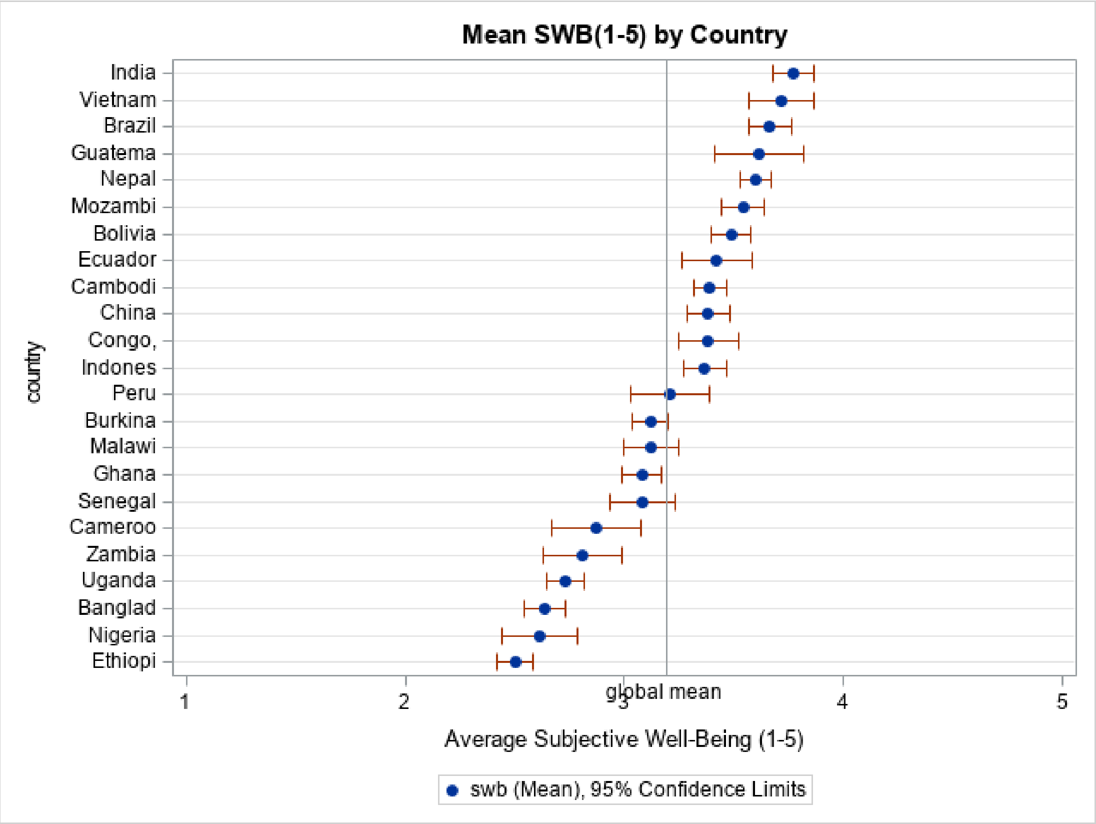


**Figure S3. Average subjective life satisfaction scores.** Subjective well-being measured on a five-point scale among forest-users of 23 countries of the PEN study. Bars reflect 95% CI.

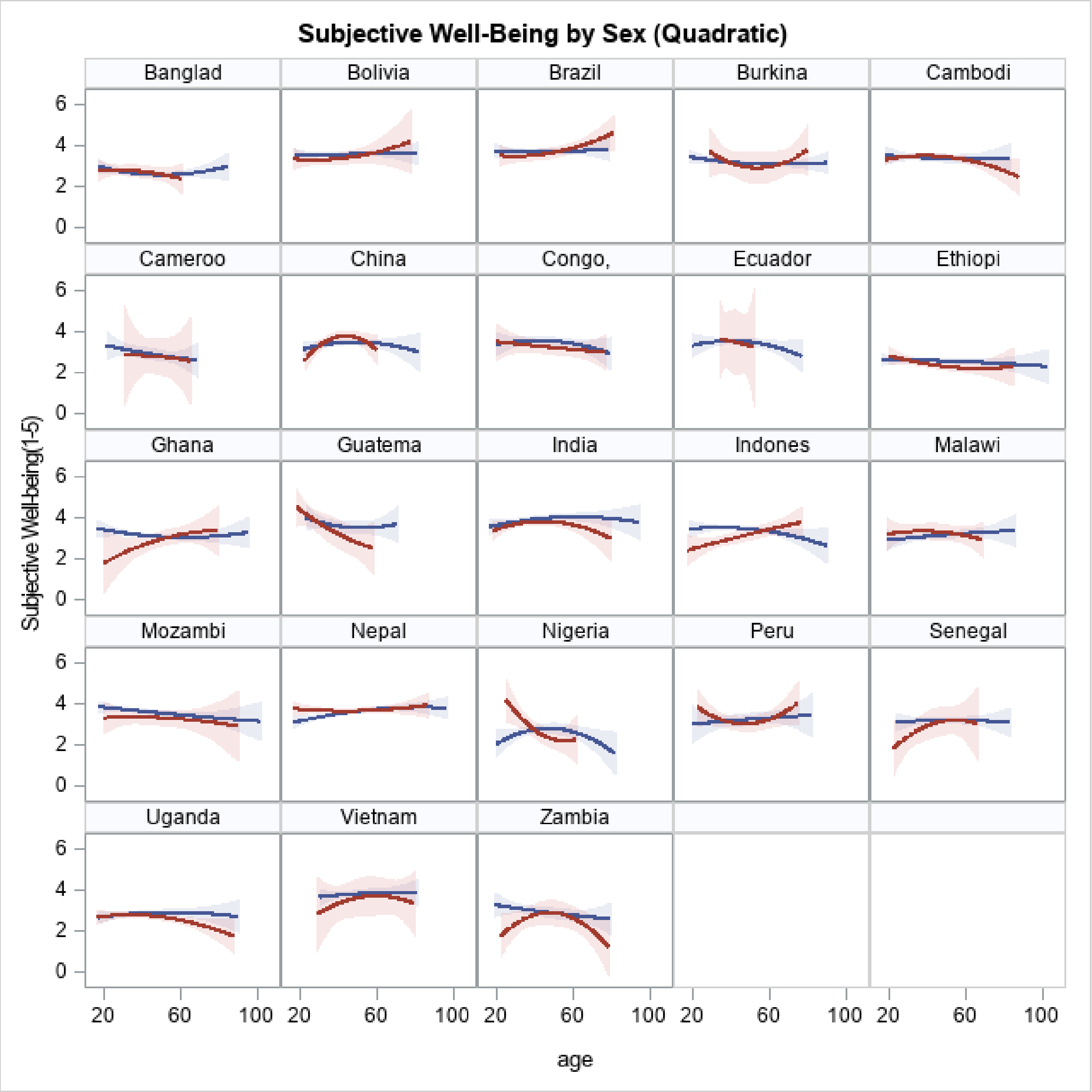


**Figure S4. Subjective life satisfaction in PEN sample of 23 countries, by sex.** Second order OLS regression of satisfaction by age. Red lines refer to females, blue lines to males.

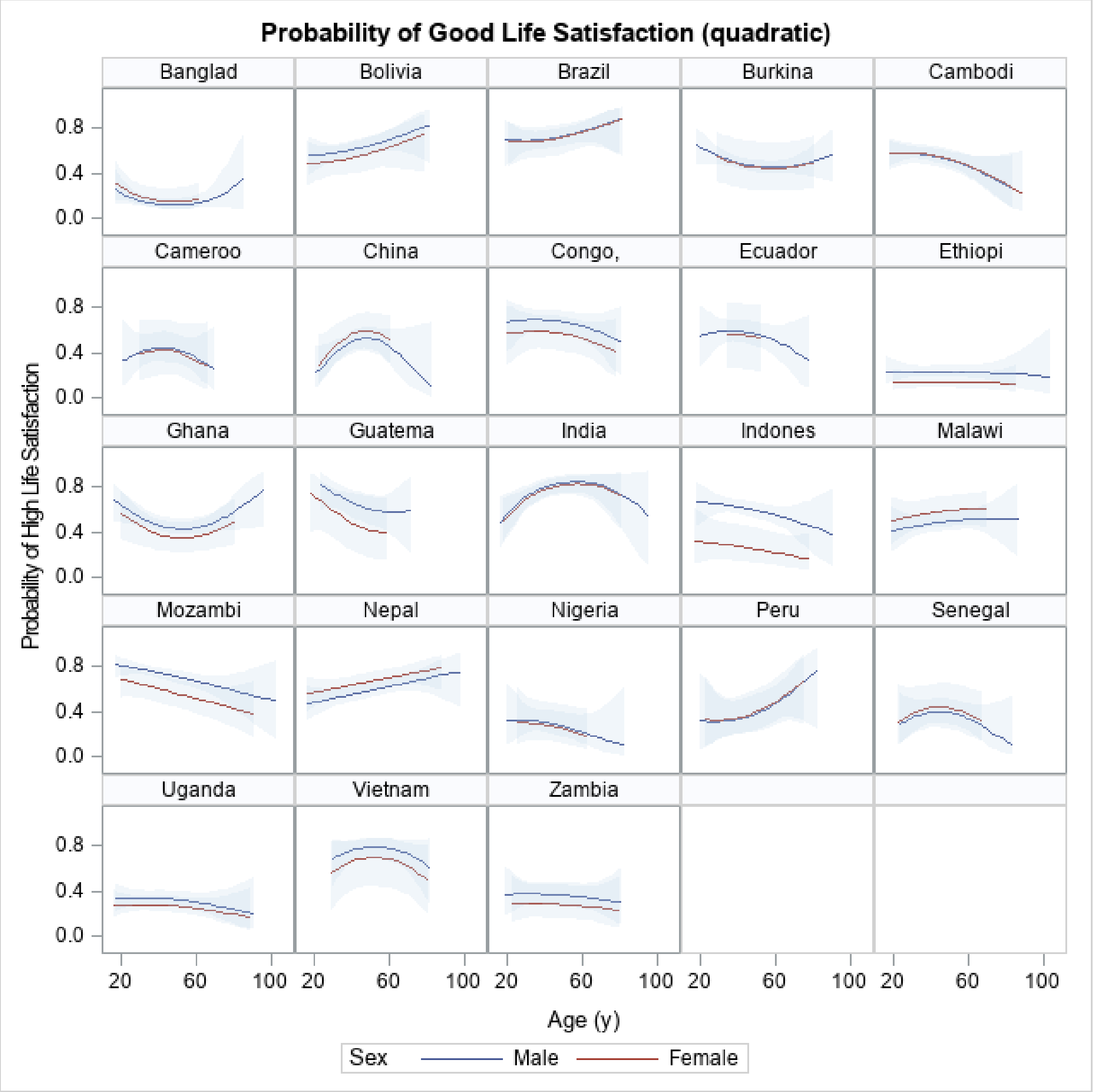


**Figure S5. Probability that subjective life satisfaction is "good".** Good is defined as 4 or 5 on the five-point scale. Model predictions (and 95% CI) from a logistic regression with age and age-squared terms. Age profiles are statistically significant at $p<0.1$ for the following countries: Bangladesh, China, Ghana, and India.

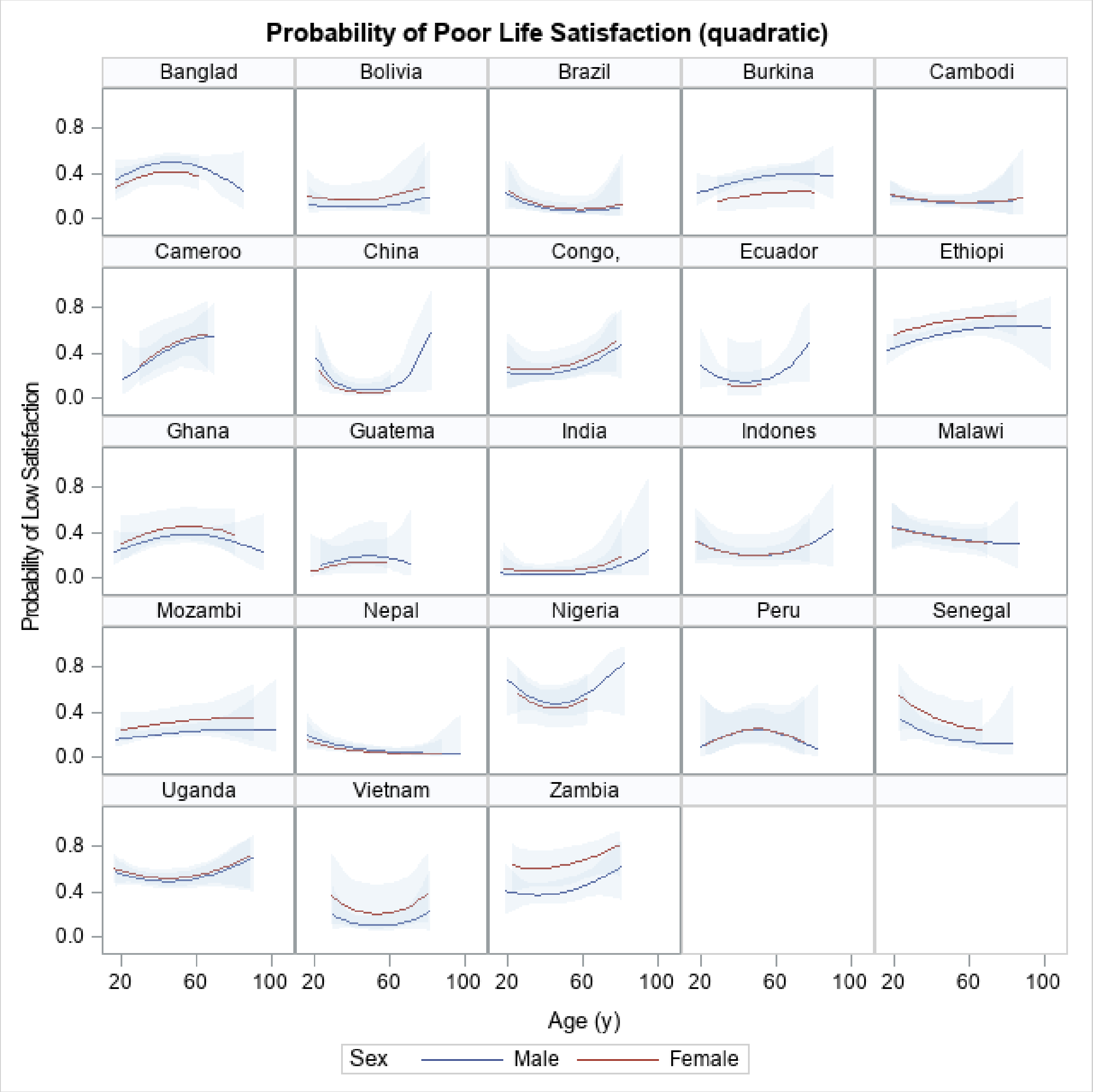


**Figure S6. Probability that subjective life satisfaction is "low".** Low is defined as 1 or 2 on the five-point scale. Model predictions (and 95% CI) from a logistic regression with age and age-squared terms. Age profiles are statistically significant at p<0.1 for the following countries: Bangladesh, China.

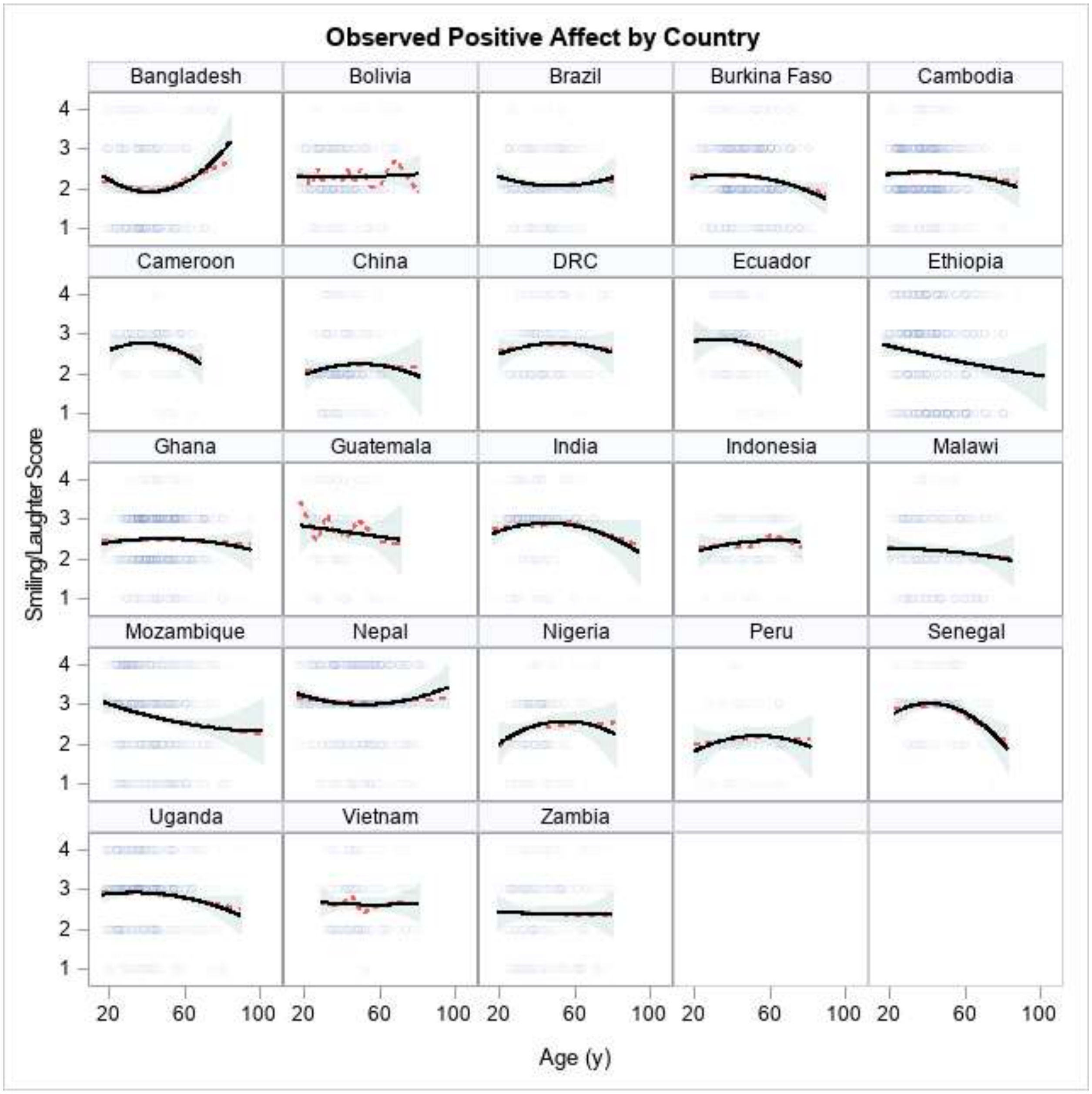


**Figure S7. Positive affect in PEN Study, by country.** Rated by interviewers for the presence of smiling and laughing, on a four-point scale, where 1=minimal smiling or laughing,…, 4=pervasive smiling and laughing. Spearman correlation between SWB and Affect score *r*=0.17, p<0.0001 (n=6714).

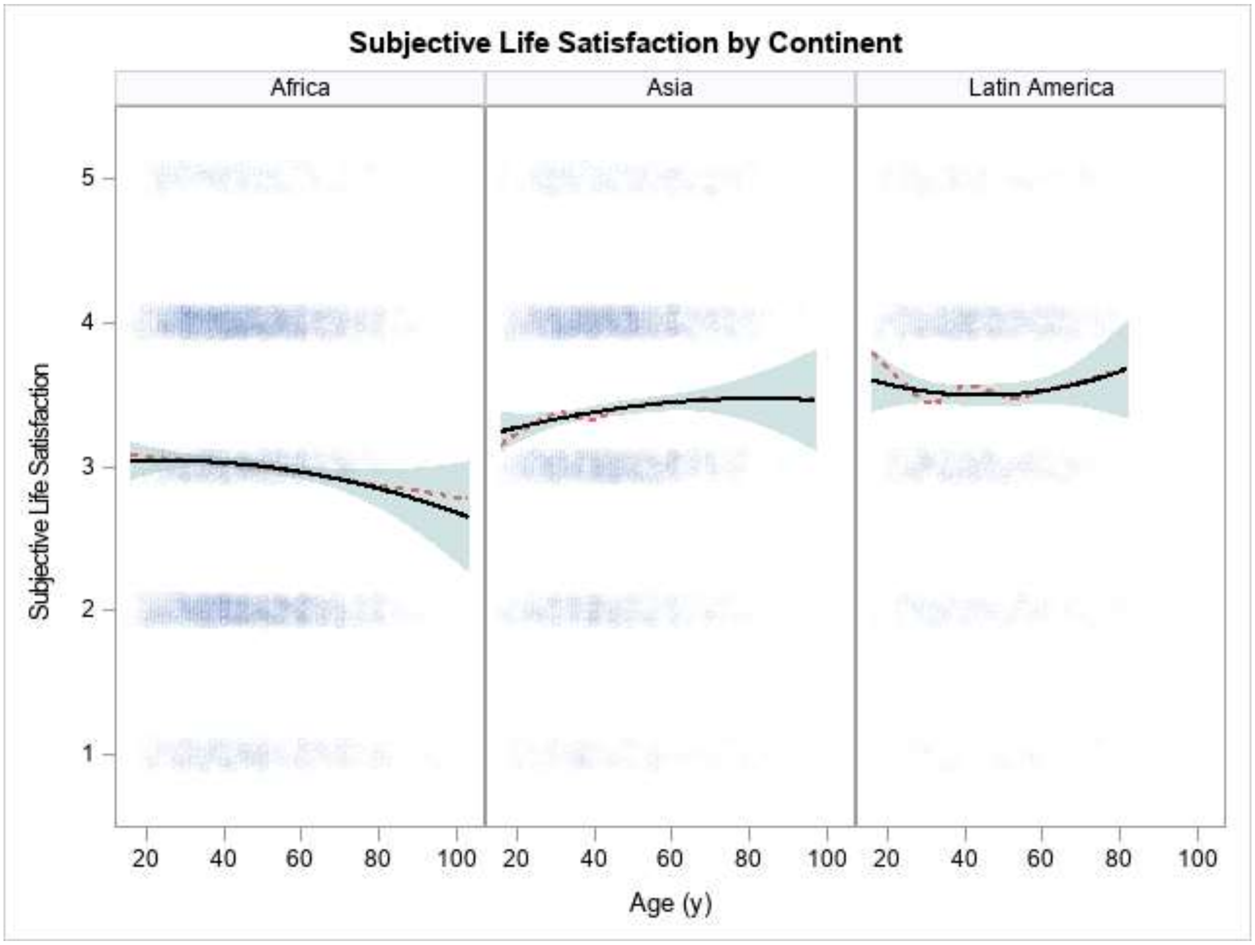


**Figure S8. Subjective life satisfaction by continent.** Loess smooth shown (in red) and OLS using age and quadratic age term (in green). Country is a random intercept.

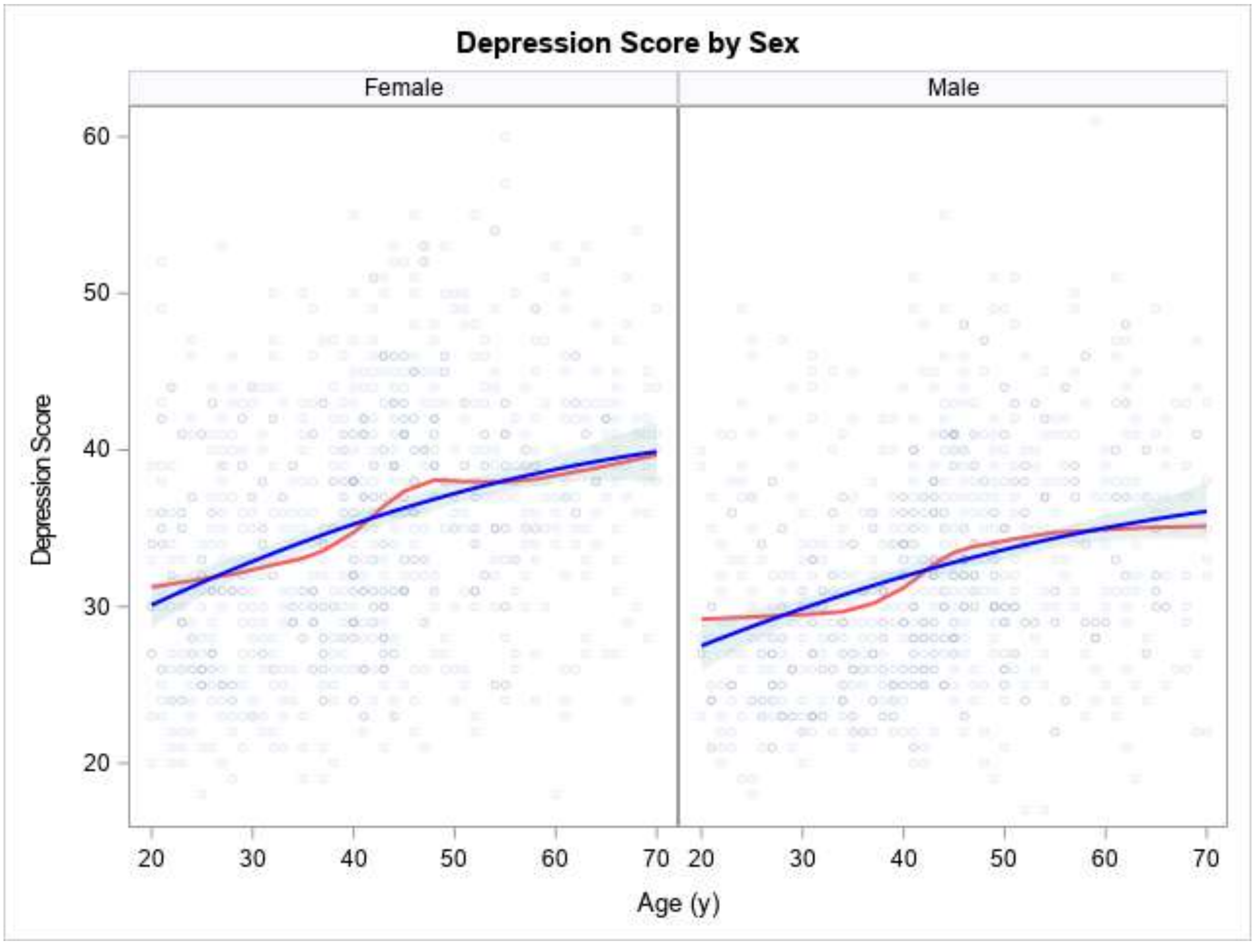


**Figure S9. Depressed affect score by sex, ages restricted to ≤ 70 y.** Trendlines are from loess and OLS regression including age (b=0.380, p<0.0001) and age$^2$ (b=-0.00215, p=0.022), $R^2$=0.15.

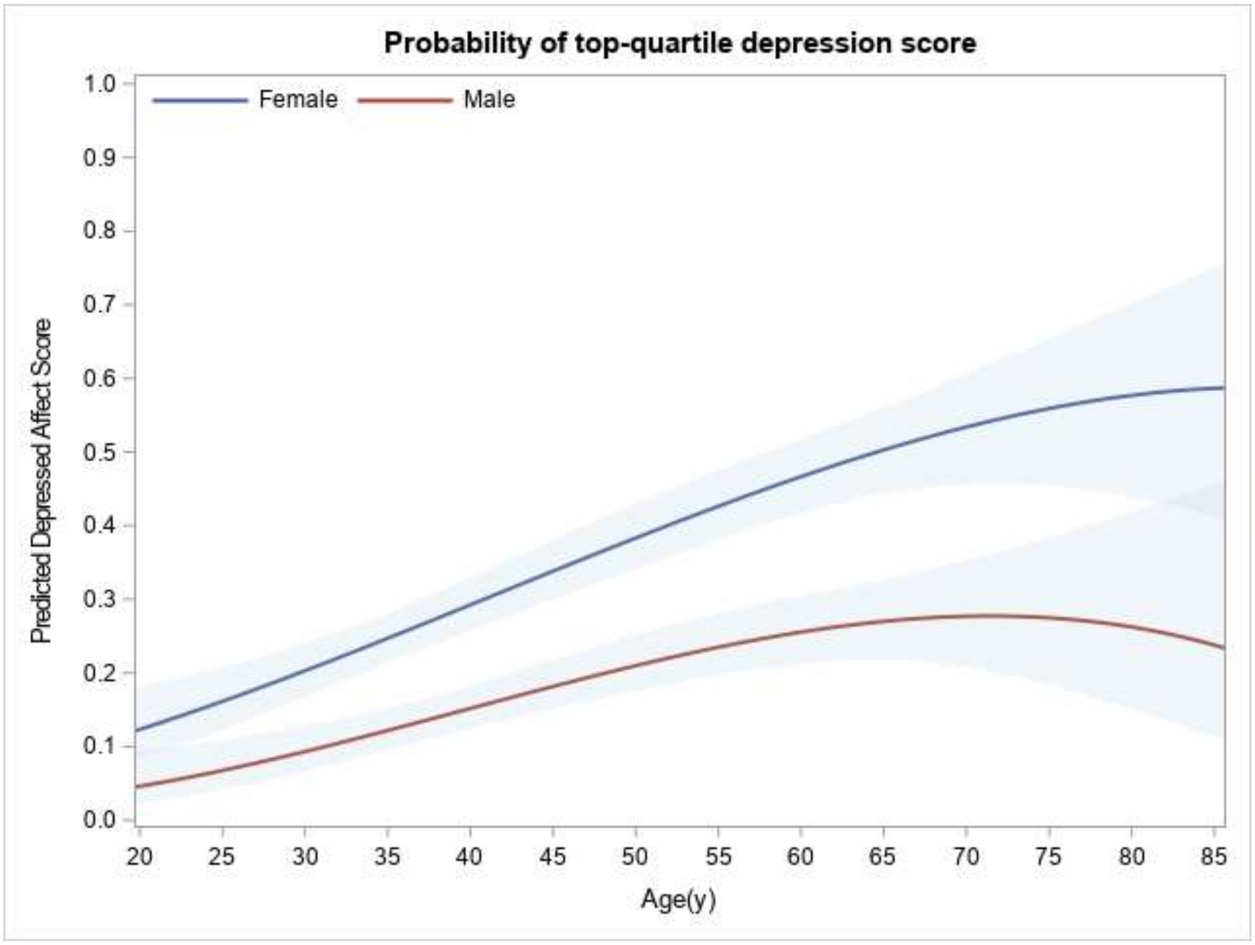


**Figure S10. Probability of Depressed affect score in top-quartile, by sex.** Logistic regression on cross-sectional sample.

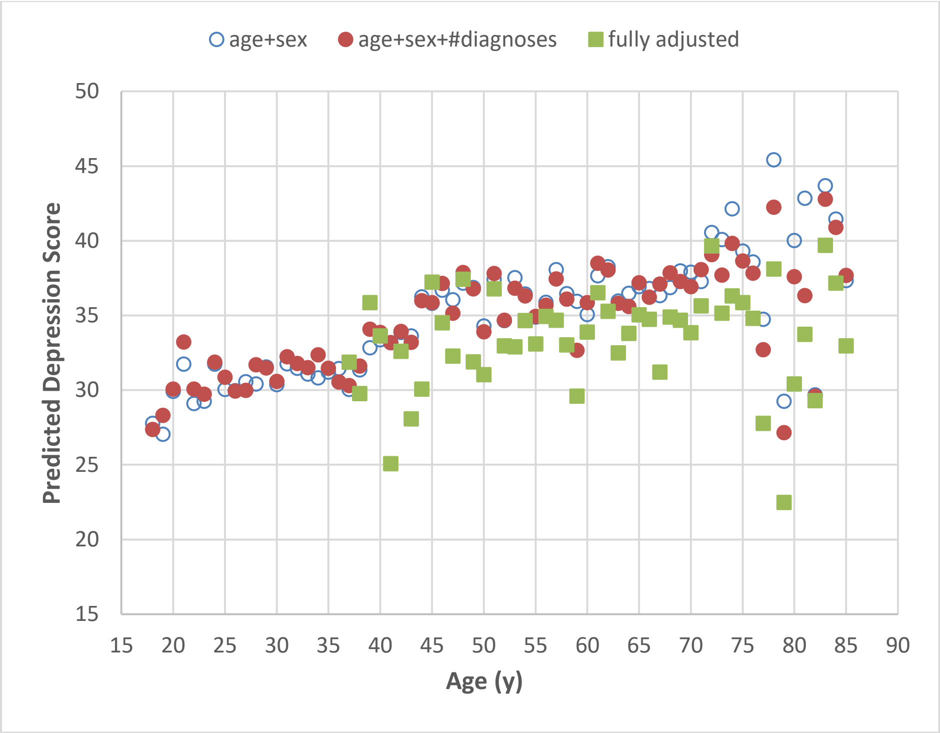


**Figure S11. Predicted depression score among Tsimane', using Age as a categorical variable.** OLS regression. Model 1 (open circles) adjusts for sex. Model 2 (solid circles) additionally adjusts for the number of physician-based diagnoses, grouped into macro-categories using International Classification of Diseases (ICD10). Model 3 (grey squares), restricted to ages 37+ y, additionally adjusts for activities of daily living (ADLs), self-reports of physical pain (yes/no), problems with daily activities (yes/no), problems seeing, and health belief score (see Methods). Covariates evaluated at population mean.

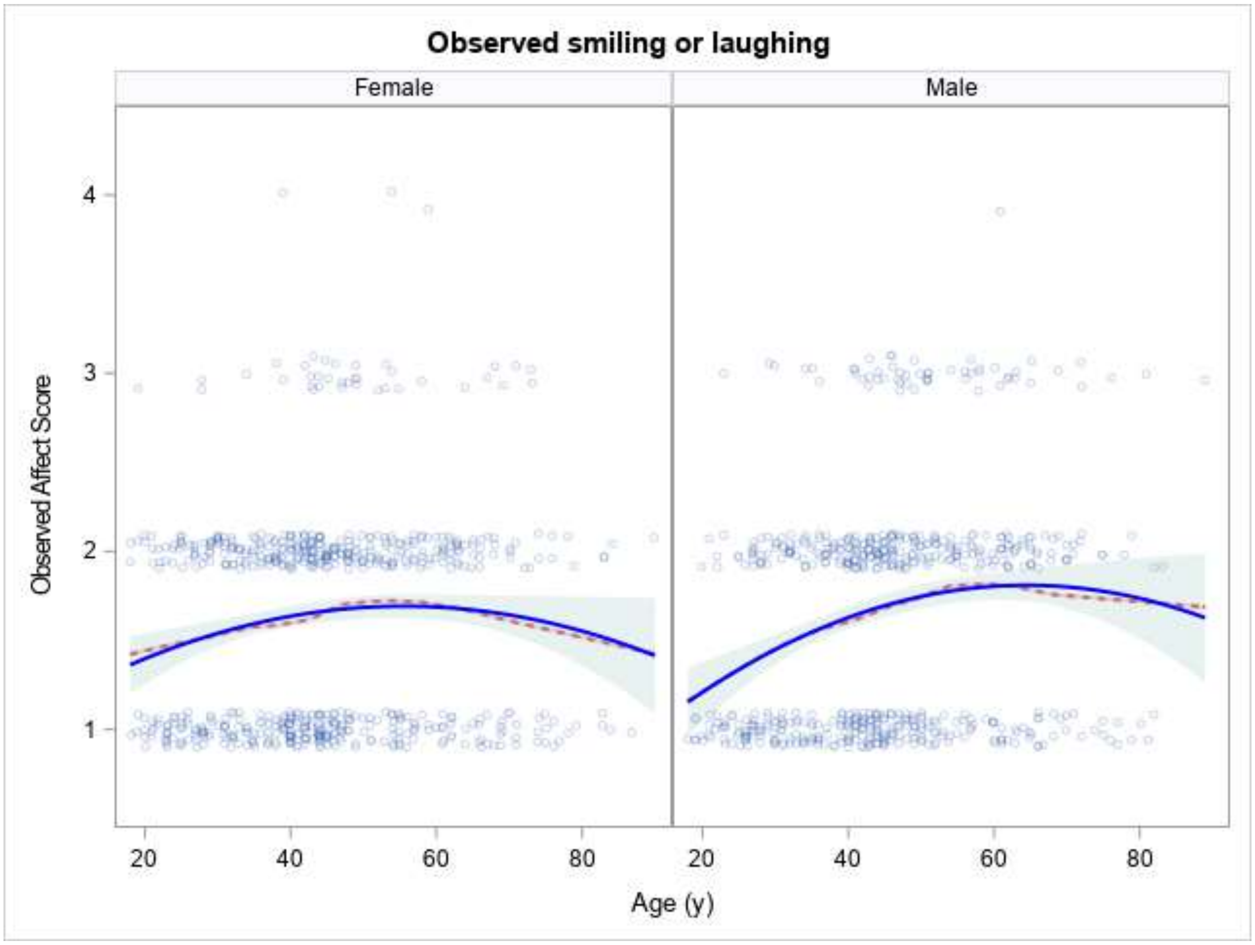


**Figure S12. Happy affect score by observer.** Tsimane' interviewers rated participants after a series of interviews. Scoring is: 1=didn't smile or laugh once, 2=didn't laugh but smiled, 3=smiled and laughed a few times, 4=laughed and smiled often. From OLS regression (n=1204): age: b=0.032, p<0.0001; age2: b=-2.64E-4, p=0.0002; $R^2$=0.03. Number of diagnoses is also positively associated with affect score when added to this baseline model (b=0.066, p<0.0001). In full model including all the health-related variables of Model 3 in Table S9, age effects are substantially reduced (age: b=-0.00633, p=0.855; age$^2$: b=1.56E-6, p=0.996).

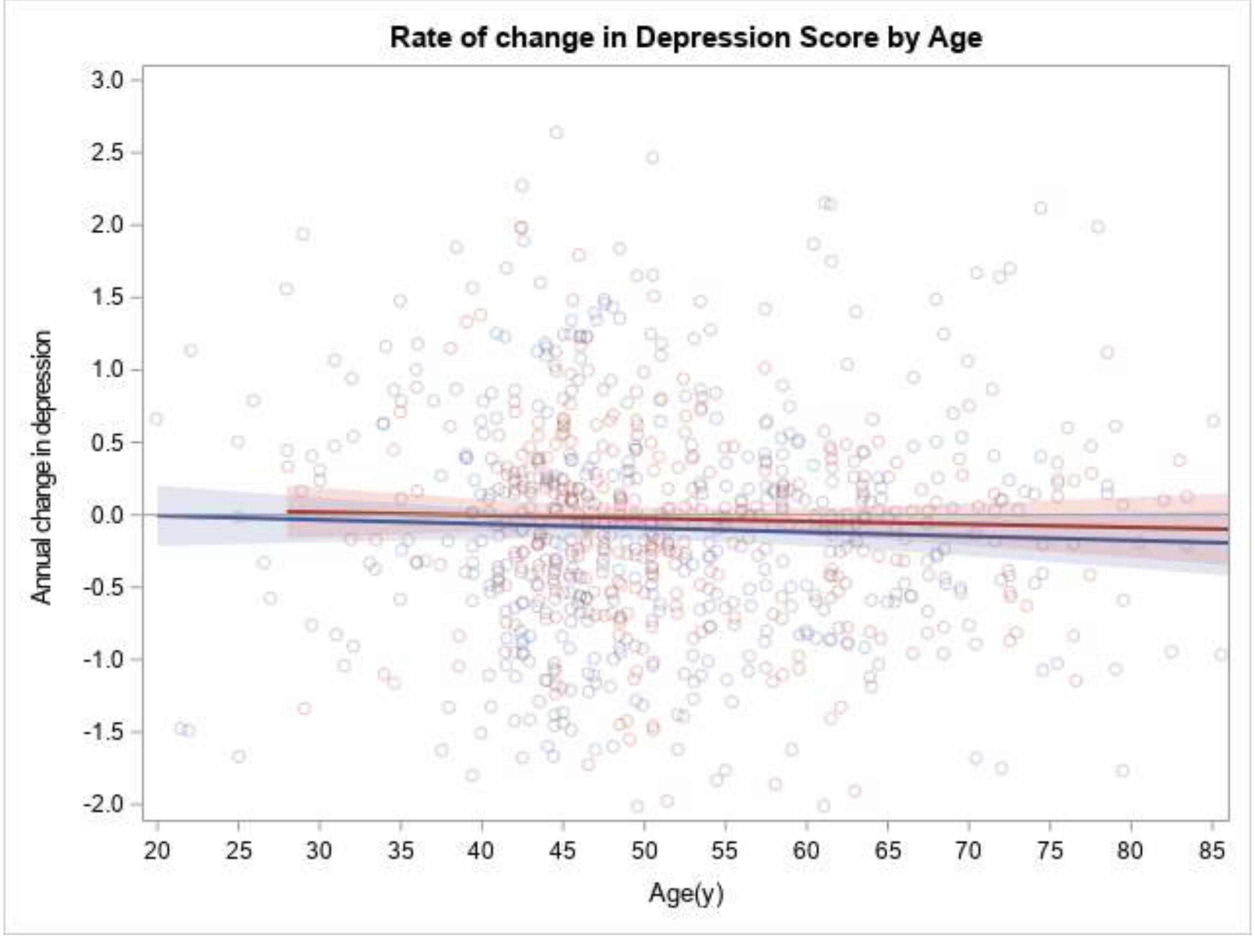


**Figure S13. Rate of change in depression score by age and sex.** Rate of change in depression score, *D*, for adult aged *t* is calculated as $(D_{t+g}-D_t)$/g, where *D* is a normalized z-scored depression score, *g* is the gap (in years) between sampling periods. Average±SD for *g* is 2.23±1.35 (range 0.64 to 7.52 years). We eliminate outliers by restricting annual rate of change to <|4| and *g*>0.5. In OLS regression adjusting for sex, rate of change is not significantly related to age (b=-0.00253, p=0.280), crossing the x-axis at age 27 y. In a full model adjusting for number of diagnoses at time 1, change in number of diagnoses, physical pain at time 1, change in physical pain rating, problems in productive activities, change in problems with activities, health belief score at time 1, and change in health belief score, the effect of age remains relatively unchanged (b=-0.0045, p=0.127).

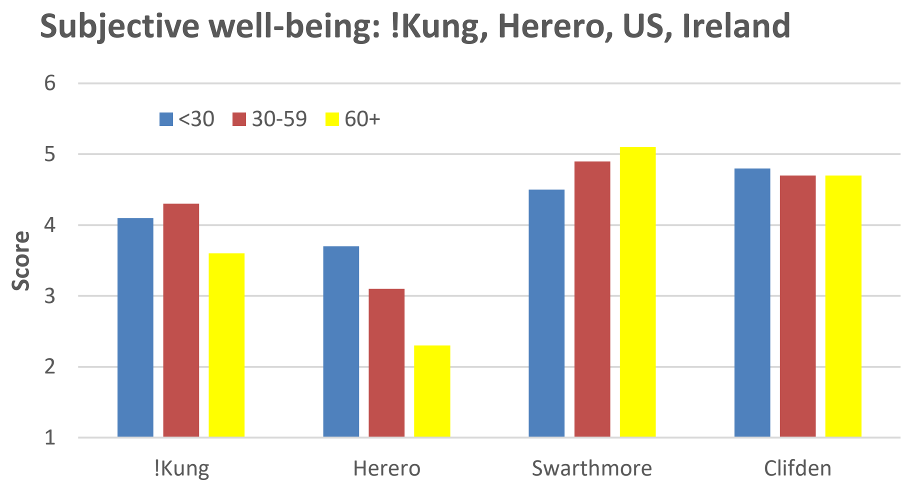


**Figure S14. Subjective well-being from Project AGE.** !Kung (n=102), Herero (n=186), Swarthmore, PA (n=201), Ireland (n=129). From Keith et al. 1994 (Ch. 5: Table 5.1). Well-being was defined as "assessment of one's own life compared to one's definitions of the best life possible", and evaluated as a modified Cantril Self-Anchoring Ladder. The ladder was measured on five-point scale for !Kung and Herero, but adapted to a six-point scale by the team to facilitate cross-cultural comparisons.

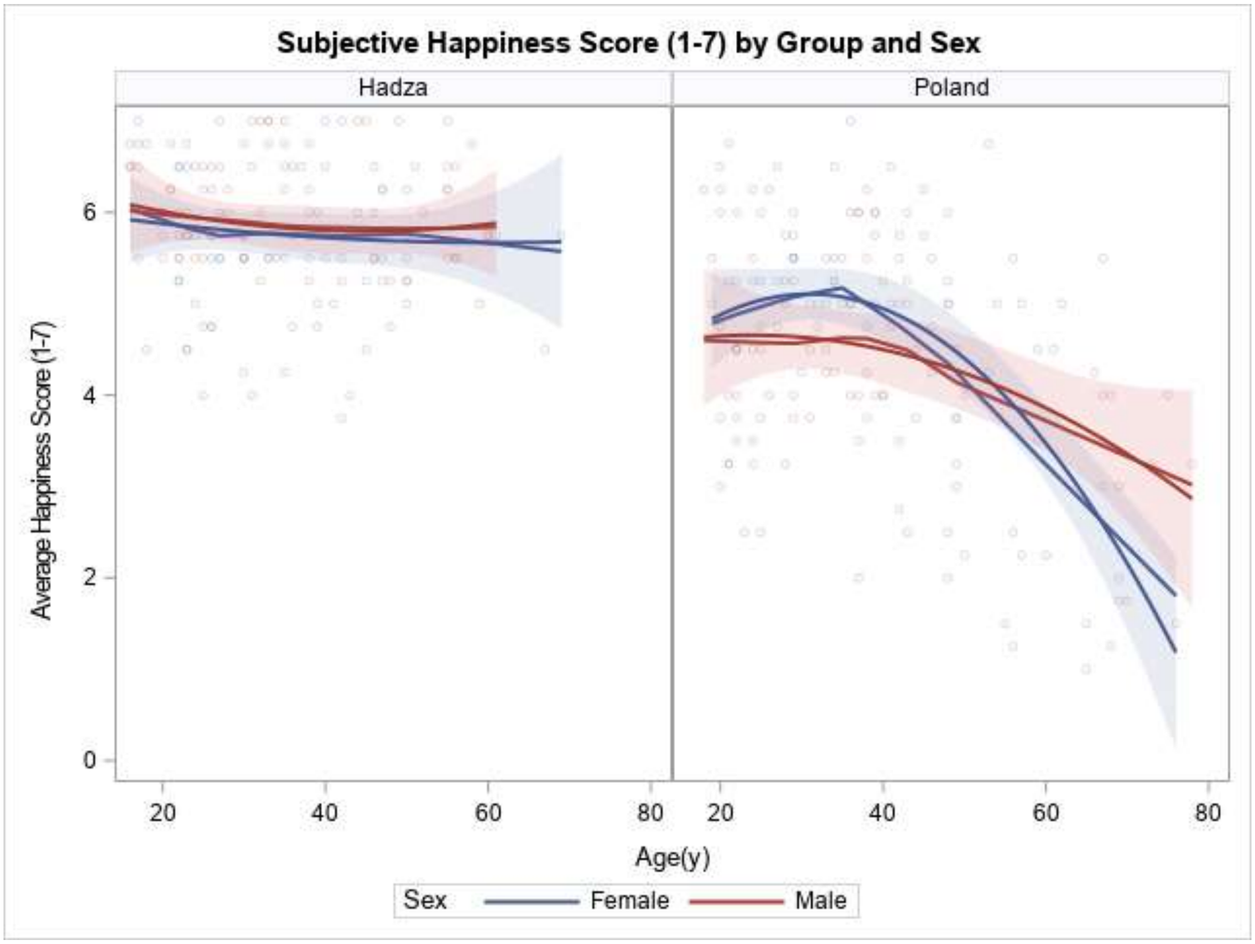


**Figure S15. Subjective happiness among Hadza (n=145) and Polish (n=156).** Happiness score is average of 7-point scale from four questions: 1) In general I consider myself (1=not a very happy person, 7=a very happy person); 2) Compared to most of my peers, I consider myself (1=less happy, 7=more happy); 3) Some people are generally very happy. They enjoy life regardless of what is going on, getting the most out of everything. To what extent does this characterization describe you? (1=not at all, 7=a great deal); 4) Some people are generally not very happy. Although they are not depressed, they never seem as happy as they might be. To what extent does this characterization describe you? (1=a great deal, 7=not at all). Correlations of the average score with each of the four questions are ≥0.57 for Hadza and ≥0.86 for Poland. Hadza recruited in 2018 from nine camps in rural, northern Tanzania around Lake Eyasi. Polish participants were recruited in a "provincial town in South-West Poland during educational courses at the local University". From Frankowiak et al. 2020 (Reanalyzed from data made public).

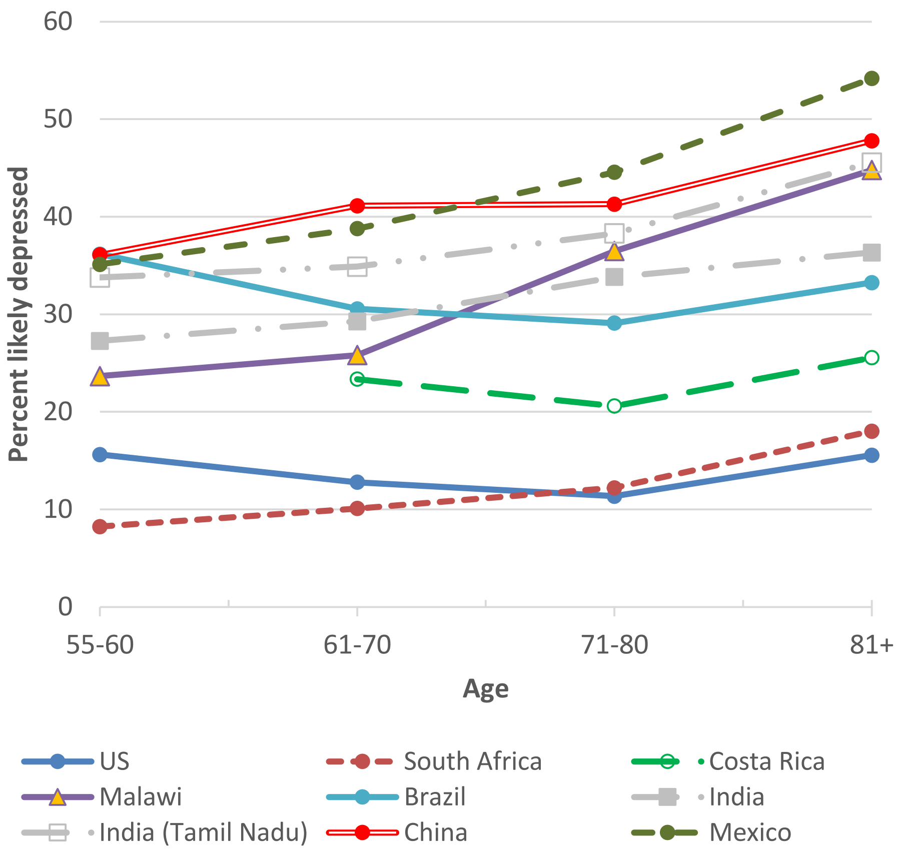


**Figure S16. Prevalence of depression in low- and middle-income countries.** Averages men and women. United States and Brazil profile consistent with the U-shaped happiness curve. All other countries show evidence of greater depression symptoms with age. All data are from nationally representative samples of older adults from each country, except India (Tamil Nadu). See Banerjee et al. (2023) for details on sources and methods.

| Society | n (obs/inds) | Age (y) Mean | Age STD | % female |
|---|---|---|---|---|
| Baka | 460/223 | 36.4 | 15.0 | 55.7% |
| Punan | 309/110 | 36.6 | 14.5 | 50.8% |
| Tsimane’ | 405/135 | 36.3 | 18.1 | 48.8% |
| Total | 1174/468 | 36.4 | 16.0 | 52.0% |

**Table S1. Baka, Punan and Tsimane’ Sample Descriptives.**

| | Baka | | | | | | Punan | | | | | | Tsimane | | | | | |
|---|---|---|---|---|---|---|---|---|---|---|---|---|---|---|---|---|---|---|
| | Model 1A: Age | | | Model 2A: Age, $Age^2$ | | | Model 1B: Age | | | Model 2B: Age, $Age^2$ | | | Model 1C: Age | | | Model 2C: Age, $Age^2$ | | |
| **Effect** | **est.** | **s.e.** | **Pr > \|t\|** | **est.** | **s.e.** | **Pr > \|t\|** | **est.** | **s.e.** | **Pr > \|t\|** | **est.** | **s.e.** | **Pr > \|t\|** | **est.** | **s.e.** | **Pr > \|t\|** | **est.** | **s.e.** | **Pr > \|t\|** |
| ***OLS Regression*** | | | | | | | | | | | | | | | | | | |
| **age** | *-0.006* | 0.003 | 0.051 | **-0.051** | 0.018 | 0.005 | **-0.014** | 0.005 | 0.008 | **-0.061** | 0.022 | 0.006 | **-0.009** | 0.003 | 0.001 | -0.012 | 0.012 | 0.332 |
| **$age^2$** | | | | **0.00054** | 0.0002 | 0.012 | | | | **0.00053** | 0.000235 | 0.026 | | | | 2.6E-05 | 0.00013 | 0.834 |
| female | -0.076 | 0.091 | 0.405 | -0.109 | 0.091 | 0.234 | -0.306 | 0.148 | 0.040 | -0.343 | 0.146 | 0.020 | -0.168 | 0.101 | 0.096 | -0.169 | 0.101 | 0.097 |
| | | | | | | | | | | | | | | | | | | |
| **50+** | 0.186 | 0.139 | 0.182 | | | | 0.028 | 0.209 | 0.895 | | | | *-0.295* | 0.166 | 0.077 | | | |
| **<35** | **0.282** | 0.113 | 0.013 | | | | **0.555** | 0.162 | 8E-04 | | | | 0.048 | 0.117 | 0.681 | | | |
| **35-49** | ref | . | . | | | | ref | . | . | | | | ref | . | . | | | |
| female | -0.106 | 0.093 | 0.255 | | | | -0.292 | 0.144 | 0.044 | | | | -0.168 | 0.103 | 0.105 | | | |
| | | | | | | | | | | | | | | | | | | |
| **Ordered Logistic Regression** | | | | | | | | | | | | | | | | | | |
| **age** | -0.017 | 0.0085 | 0.047 | **-0.1417** | 0.055 | 0.01 | **-0.03** | 0.011 | 0.005 | **-0.1325** | 0.04733 | 0.006 | **-0.019** | 0.006 | 0.002 | -0.0307 | 0.02629 | 0.244 |
| **$age^2$** | | | | **0.00147** | 6E-04 | 0.023 | | | | **0.00113** | 0.000506 | 0.026 | | | | 0.00013 | 0.00026 | 0.636 |
| female | -0.22 | 0.2618 | 0.403 | -0.3099 | 0.266 | 0.245 | -0.577 | 0.312 | 0.066 | -0.6637 | 0.3132 | 0.035 | -0.347 | 0.216 | 0.11 | -0.3516 | 0.2169 | 0.106 |

**Table S2. OLS Regression of Subjective Life Satisfaction among Baka, Punan and Tsimane'.** For each population, models are shown that consider linear age effect, age and $age^2$, and age categories (<35 y, 35-49, 50+).

| | Model 1: Age | | | Model 2: Age, $Age^2$ | | | Model 3: Age, $Age^2$, Pop'n | | | Model 4: Full model | | |
|---|---|---|---|---|---|---|---|---|---|---|---|---|
| **Effect** | **est.** | **s.e.** | **Pr > \|t\|** | **est.** | **s.e.** | **Pr > \|t\|** | **est.** | **s.e.** | **Pr > \|t\|** | **est.** | **s.e.** | **Pr > \|t\|** |
| **Age** | **-0.009** | 0.002 | <.0001 | **-0.027** | 0.009 | 0.004 | **-0.028** | 0.009 | 0.002 | -0.011 | 0.013 | 0.381 |
| **$Age^2$** | | | | *0.0002* | 0.0001 | 0.0514 | **0.00021** | 9.7E-05 | 0.03 | 2.1E-05 | 0.00013 | 0.8772 |
| female | -0.137 | 0.066 | 0.038 | -0.145 | 0.066 | 0.028 | -0.170 | 0.063 | 0.007 | -0.188 | 0.063 | 0.003 |
| Population: | | | | | | | | | | | | |
| Baka | | | | | | | **0.308** | 0.074 | <.0001 | **0.962** | 0.452 | 0.034 |
| Punan | | | | | | | **-0.204** | 0.083 | 0.014 | 0.722 | 0.465 | 0.121 |
| Pop'n*Age | | | | | | | | | | | | |
| Baka | | | | | | | | | | *-0.041* | 0.023 | 0.075 |
| Punan | | | | | | | | | | **-0.046** | 0.022 | 0.039 |
| Pop'n*$Age^2$ | | | | | | | | | | | | |
| Baka | | | | | | | | | | **0.00054** | 0.00026 | 0.040 |
| Punan | | | | | | | | | | **0.00048** | 0.00024 | 0.046 |

**Table S3. OLS Regression of Combined Sample, adding populations and interaction terms.**

| | Baka | | | Punan | | | Tsimane | | | All groups | | |
|---|---|---|---|---|---|---|---|---|---|---|---|---|
| | **Model 1** | | | **Model 2** | | | **Model 3** | | | **Model 4** | | |
| **Effect** | **est.** | **s.e.** | **Pr > \|t\|** | **est.** | **s.e.** | **Pr > \|t\|** | **est.** | **s.e.** | **Pr > \|t\|** | **est.** | **s.e.** | **Pr > \|t\|** |
| Age (y) | **-0.049** | 0.021 | 0.022 | **-0.055** | 0.025 | 0.026 | 0.002 | 0.013 | 0.878 | **-0.023** | 0.010 | 0.029 |
| Age$^2$ | **5.54E-04** | 2.42E-04 | 0.023 | *4.49E-04* | 2.57E-04 | 0.083 | -1.10E-04 | 1.33E-04 | 0.416 | *1.84E-04* | 1.09E-04 | 0.091 |
| Female | -0.122 | 0.109 | 0.266 | -0.158 | 0.161 | 0.327 | -0.112 | 0.107 | 0.295 | *-0.131* | 0.072 | 0.070 |
| *Confounders* | | | | | | | | | | | | |
| Highest Level Schooling | -0.019 | 0.050 | 0.700 | -0.017 | 0.035 | 0.640 | 0.014 | 0.034 | 0.684 | 0.012 | 0.022 | 0.599 |
| Household Size | 0.028 | 0.018 | 0.122 | -0.003 | 0.032 | 0.921 | **-0.077** | 0.023 | 0.001 | 0.013 | 0.013 | 0.317 |
| *Mediators* | | | | | | | | | | | | |
| Total wages (PPP) | 2.97E-03 | 7.13E-03 | 0.677 | **4.75E-03** | 1.81E-03 | 0.010 | -1.40E-04 | 1.66E-04 | 0.384 | -7.00E-05 | 1.89E-04 | 0.727 |
| Household wealth (PPP) | -2.50E-04 | 6.71E-04 | 0.714 | 1.70E-05 | 3.10E-05 | 0.571 | 2.70E-05 | 2.50E-05 | 0.277 | **-4.00E-05** | 1.60E-05 | 0.005 |
| Sick days/no work | **-0.066** | 0.024 | 0.006 | **-0.101** | 0.040 | 0.012 | -0.075 | 0.057 | 0.192 | **-0.092** | 0.020 | <.0001 |

**Table S4. OLS Regression of Subjective Life Satisfaction among Baka, Punan and Tsimane', with covariates.** Compare these models against those including just age, age$^2$ and sex (Models 2A, 2B, 2C of Table S2, and Model 2 of Table S3).

| Country | n | Age (y) Mean | Age STD | % female | Household Size | % married |
|---|---|---|---|---|---|---|
| Bangladesh | 358 | 41.0 | 14.0 | 21.2 | 4.9 | 95.0 |
| Bolivia | 285 | 40.2 | 14.1 | 20.0 | 5.7 | 89.5 |
| Brazil | 241 | 44.5 | 13.7 | 24.1 | 5.5 | 88.8 |
| Burkina Faso | 554 | 49.8 | 14.4 | 3.4 | 10.0 | 97.3 |
| Cambodia | 544 | 39.2 | 12.8 | 73.9 | 5.5 | 90.8 |
| Cameroon | 109 | 44.7 | 11.6 | 13.8 | 6.2 | 88.1 |
| China | 227 | 41.6 | 10.7 | 15.4 | 4.7 | 91.6 |
| Congo | 178 | 45.6 | 13.6 | 27.5 | 4.9 | 74.7 |
| Ecuador | 150 | 43.7 | 11.8 | 6.0 | 6.6 | 90.7 |
| Ethiopia | 565 | 44.3 | 15.4 | 20.4 | 7.2 | 88.1 |
| Ghana | 570 | 47.0 | 13.5 | 6.5 | 6.4 | 90.5 |
| Guatemala | 109 | 40.6 | 12.8 | 17.4 | 4.7 | 96.3 |
| India | 239 | 40.8 | 13.5 | 53.1 | 4.9 | 88.3 |
| Indonesia | 354 | 44.9 | 13.5 | 8.8 | 4.7 | 90.7 |
| Malawi | 265 | 45.4 | 14.7 | 23.4 | 5.7 | 88.7 |
| Mozambique | 534 | 43.2 | 15.3 | 12.5 | 6.1 | 88.0 |
| Nepal | 487 | 48.6 | 16.8 | 34.5 | 6.0 | 78.4 |
| Nigeria | 146 | 42.6 | 11.9 | 17.8 | 6.2 | 89.7 |
| Peru | 86 | 48.0 | 14.0 | 38.4 | 5.3 | 89.5 |
| Senegal | 138 | 46.6 | 13.2 | 18.1 | 10.9 | 85.5 |
| Uganda | 506 | 40.0 | 14.8 | 44.9 | 6.1 | 81.2 |
| Vietnam | 155 | 49.8 | 12.8 | 12.3 | 3.8 | 89.0 |
| Zambia | 187 | 44.7 | 15.3 | 21.4 | 5.7 | 80.7 |
| **Continent** | | | | | | |
| Africa | 3752 | 45.0 | 14.7 | 18.2 | 7.0 | 87.9 |
| Asia | 2364 | 43.4 | 14.4 | 36.3 | 5.1 | 88.6 |
| Latin America | 871 | 42.8 | 13.7 | 20.2 | 5.6 | 90.4 |
| **Total** | 6987 | 44.2 | 14.5 | 24.6 | 6.2 | 88.4 |

**Table S5. Poverty Environmental Network (PEN) sample descriptives.** PEN sample reflects forest-user sites from 23 countries.

| | Latin America | | | Asia | | | Africa | | |
|---|---|---|---|---|---|---|---|---|---|
| **Variables** | **est.** | **s.e.** | **p** | **est.** | **s.e.** | **p** | **est.** | **s.e.** | **p** |
| *Baseline model* | | | | | | | | | |
| **Age (y)** | -0.012 | 0.013 | 0.323 | 0.007 | 0.007 | 0.307 | -0.002 | 0.006 | 0.743 |
| **Age$^2$** | 1.42E-04 | 1.36E-04 | 0.297 | -6.00E-05 | 6.90E-05 | 0.393 | -2.00E-05 | 6.40E-05 | 0.711 |
| **Sex (1=female)** | -0.074 | 0.075 | 0.318 | -0.012 | 0.043 | 0.790 | **-0.164** | 0.046 | 0.000 |
| | | | | | | | | | |
| *Full model* | | | | | | | | | |
| **Age (y)** | *-0.024* | 0.013 | 0.071 | 0.005 | 0.006 | 0.403 | -0.003 | 0.006 | 0.653 |
| **Age$^2$** | *2.40E-04* | 1.39E-04 | 0.084 | -2.00E-05 | 6.60E-05 | 0.759 | -5.40E-07 | 6.30E-05 | 0.993 |
| **Sex (1=female)** | -0.066 | 0.076 | 0.385 | 0.032 | 0.042 | 0.446 | -0.034 | 0.052 | 0.516 |
| **Absolute income** | 0.000 | 0.000 | 0.692 | **0.000** | 0.000 | 0.002 | **0.000** | 0.000 | 0.014 |
| **Relative income** | -0.009 | 0.065 | 0.885 | **0.103** | 0.027 | 0.000 | **0.182** | 0.021 | <.0001 |
| **Education (1=any)** | -0.036 | 0.087 | 0.682 | **0.116** | 0.040 | 0.004 | 0.044 | 0.042 | 0.303 |
| **Household Size** | 0.017 | 0.012 | 0.154 | **0.035** | 0.009 | <.0001 | **0.027** | 0.005 | <.0001 |
| **Married (1=yes)** | -0.157 | 0.105 | 0.133 | **0.121** | 0.057 | 0.033 | *0.106* | 0.059 | 0.075 |
| **Illness/Death** | **-0.286** | 0.091 | 0.002 | **-0.328** | 0.052 | <.0001 | **-0.118** | 0.043 | 0.007 |
| **Crop/Animal Loss** | -0.145 | 0.090 | 0.107 | **-0.227** | 0.053 | <.0001 | -0.067 | 0.049 | 0.170 |
| **Other Shocks** | 0.098 | 0.107 | 0.358 | 0.009 | 0.061 | 0.885 | **-0.133** | 0.061 | 0.029 |
| **Community Help** | -0.062 | 0.040 | 0.122 | **0.194** | 0.032 | <.0001 | **0.106** | 0.026 | <.0001 |
| **Community Trust** | **0.232** | 0.049 | <.0001 | **0.137** | 0.036 | 0.000 | **0.156** | 0.031 | <.0001 |

**Table S6. OLS regression of subjective life satisfaction in PEN sample, by continent.** Country included as random effect. *Absolute Income* is household income scaled by adult equivalent units and *Relative income* scales to purchasing power exchange rates. *Married* (1=yes, 0=no, referring to being single, divorced or widowed); For shocks, *Illness/Death* refers to and illness or death experienced by household members in the last year. *Other Shocks* refers to whether the household faced any major crisis or unexpectedly large expenditures in the past year. *Crop/Animal Loss* refers to whether household suffered any major loss of crops or livestock; *Help* is response to question: ‘‘Can you get help from other people in the village if you are in need, for example, if you need extra money because someone in your family is sick?’’ (1 = yes, 0 = no or sometimes, but not always; *Trust* is response to: ‘‘In general, do you trust people in the village?’’ (1 = yes; 0 = no or I trust some people but not others).

| Variables | Latin America est. | Latin America s.e. | Latin America p | Asia est. | Asia s.e. | Asia p | Africa est. | Africa s.e. | Africa p |
|---|---|---|---|---|---|---|---|---|---|
| *Baseline model* | | | | | | | | | |
| **Age (y)** | -0.008 | 0.030 | 0.782 | 0.015 | 0.015 | 0.317 | -0.010 | 0.012 | 0.395 |
| **Age$^2$** | 1.62E-04 | 3.24E-04 | 0.617 | -1.40E-04 | 1.57E-04 | 0.360 | 3.10E-05 | 1.20E-04 | 0.798 |
| **Sex (1=female)** | -0.175 | 0.173 | 0.313 | 0.039 | 0.099 | 0.694 | **-0.282** | 0.087 | 0.001 |
| *Full model* | | | | | | | | | |
| **Age (y)** | -0.029 | 0.032 | 0.370 | 0.018 | 0.016 | 0.280 | -0.012 | 0.013 | 0.329 |
| **Age$^2$** | 3.31E-04 | 3.41E-04 | 0.333 | -1.10E-04 | 1.69E-04 | 0.521 | 7.10E-05 | 1.25E-04 | 0.572 |
| **Sex (1=female)** | -0.158 | 0.182 | 0.385 | 0.155 | 0.108 | 0.151 | -0.061 | 0.102 | 0.546 |
| **Absolute income** | 0.000 | 0.000 | 0.517 | **0.000** | 0.000 | <.0001 | **0.000** | 0.000 | 0.047 |
| **Relative income** | -0.087 | 0.164 | 0.597 | 0.079 | 0.091 | 0.386 | **0.379** | 0.047 | <.0001 |
| **Education (1=any)** | -0.115 | 0.217 | 0.596 | 0.244 | 0.102 | 0.017 | 0.034 | 0.083 | 0.684 |
| **Household Size** | 0.017 | 0.029 | 0.553 | **0.089** | 0.023 | <.0001 | **0.049** | 0.010 | <.0001 |
| **Married (1=yes)** | -0.481 | 0.268 | 0.073 | 0.351 | 0.141 | 0.013 | 0.191 | 0.118 | 0.104 |
| **Illness/Death** | **-0.667** | 0.215 | 0.002 | **-0.668** | 0.129 | <.0001 | **-0.198** | 0.085 | 0.019 |
| **Crop/Animal Loss** | -0.226 | 0.209 | 0.280 | **-0.568** | 0.133 | <.0001 | -0.099 | 0.094 | 0.296 |
| **Other Shocks** | 0.179 | 0.254 | 0.481 | -0.131 | 0.156 | 0.401 | **-0.279** | 0.119 | 0.019 |
| **Community Help** | -0.147 | 0.097 | 0.132 | **0.453** | 0.079 | <.0001 | **0.227** | 0.051 | <.0001 |
| **Community Trust** | **0.471** | 0.118 | <.0001 | **0.401** | 0.090 | <.0001 | **0.322** | 0.061 | <.0001 |

**Table S7. Ordered logistic regression of subjective life satisfaction in PEN sample, by continent.** Same as Table S6, but life satisfaction is on three-point scale.

| | Model 1: Age | | | Model 2: Age, Age2 | | | Model 3: Full | | |
|---|---|---|---|---|---|---|---|---|---|
| **OLS** | **est.** | **s.e.** | **p** | **est.** | **s.e.** | **p** | **est.** | **s.e.** | **p** |
| **Age (y)** | **-0.002** | 0.001 | 0.008 | 0.001 | 0.004 | 0.768 | -3.20E-04 | 0.004 | 0.940 |
| **Age$^2$** | | | | -4.00E-05 | 4.40E-05 | 0.421 | -4.24E-06 | 4.40E-05 | 0.923 |
| **Sex (1=female)** | **-0.097** | 0.030 | 0.001 | **-0.097** | 0.030 | 0.001 | -0.013 | 0.031 | 0.672 |
| **Absolute income** | | | | | | | **-1.00E-05** | 5.93E-06 | 0.019 |
| **Relative income** | | | | | | | **0.168** | 0.015 | <.0001 |
| **Education (1=any)** | | | | | | | **0.068** | 0.028 | 0.018 |
| **Household Size** | | | | | | | **0.026** | 0.004 | <.0001 |
| **Married (1=yes)** | | | | | | | **0.105** | 0.039 | 0.007 |
| **Illness/Death** | | | | | | | **-0.192** | 0.031 | <.0001 |
| **Crop/Animal Loss** | | | | | | | **-0.130** | 0.034 | 0.000 |
| **Other Shocks** | | | | | | | **-0.065** | 0.041 | 0.110 |
| **Community Help** | | | | | | | **0.104** | 0.018 | <.0001 |
| **Community Trust** | | | | | | | **0.163** | 0.022 | <.0001 |

| **ORDERED LOGISTIC** | **est.** | **s.e.** | **p** | **est.** | **s.e.** | **p** | **est.** | **s.e.** | **p** |
|---|---|---|---|---|---|---|---|---|---|
| **Age (y)** | **-0.003** | 0.002 | 0.036 | 0.001 | 0.009 | 0.877 | 0.000 | 0.009 | 0.993 |
| **Age2** | | | | -5.00E-05 | 9.00E-05 | 0.576 | -1.00E-05 | 9.40E-05 | 0.906 |
| **Sex (1=female)** | **-0.160** | 0.061 | 0.009 | **-0.159** | 0.061 | 0.009 | 0.003 | 0.067 | 0.969 |
| **Absolute income** | | | | | | | -2.00E-05 | 1.60E-05 | 0.166 |
| **Relative income** | | | | | | | **0.372** | 0.038 | <.0001 |
| **Education (1=any)** | | | | | | | *0.102* | 0.061 | 0.093 |
| **Household Size** | | | | | | | **0.050** | 0.009 | <.0001 |
| **Married (1=yes)** | | | | | | | **0.232** | 0.083 | 0.005 |
| **Illness/Death** | | | | | | | **-0.364** | 0.066 | <.0001 |
| **Crop/Animal Loss** | | | | | | | **-0.250** | 0.071 | 0.000 |
| **Other Shocks** | | | | | | | **-0.203** | 0.087 | 0.020 |
| **Community Help** | | | | | | | **0.236** | 0.038 | <.0001 |
| **Community Trust** | | | | | | | **0.363** | 0.046 | <.0001 |

**Table S8. OLS and Ordered logistic regressions of global PEN sample.** n=6986.

| Variable | N | Mean | SD | Min | Max |
| --- | --- | --- | --- | --- | --- |
| Age (y) | 1872 | 43.9 | 14.7 | 18 | 90 |
| Sex (1=male) | 1872 | 0.5 | 0.5 | 0 | 1 |
| # of medical diagnoses | 1580 | 2.1 | 1.2 | 0 | 6 |
| Activities of Daily Living (0-36) | 1759 | 0.2 | 1.4 | 0 | 24 |
| Health Belief Score (2-11) | 1396 | 7.5 | 1.3 | 4 | 11 |
| Physical Pain (1-4) | 1599 | 2.3 | 0.5 | 1 | 4 |
| Problems with Activities (1=yes) | 1590 | 0.7 | 0.5 | 0 | 1 |
| Can Walk All Day (1=yes) | 1004 | 0.6 | 0.5 | 0 | 1 |
| Problems Seeing (1-5) | 572 | 2.5 | 1.3 | 1 | 5 |
| Problems Hearing (1-5) | 574 | 1.8 | 1.0 | 1 | 5 |

**Table S9. Sample descriptives for Tsimane' depressed affect (Study 3).** Sample sizes are reduced for some variables where adults age 40+ were sampled, or 50+ (problems seeing and hearing).

| Predictors | Model 1: Baseline est. | Model 1: Baseline s.e. | Model 1: Baseline Pr > \|t\| | Model 2: Medical Diagnoses est. | Model 2: Medical Diagnoses s.e. | Model 2: Medical Diagnoses Pr > \|t\| | Model 3: Full Model est. | Model 3: Full Model s.e. | Model 3: Full Model Pr > \|t\| |
|---|---|---|---|---|---|---|---|---|---|
| **Age (y)** | 0.704 | 0.340 | 0.039 | 0.778 | 0.317 | 0.014 | 0.439 | 0.305 | 0.151 |
| **Age$^2$** | -4.69E-03 | 2.76E-03 | 0.090 | -5.35E-03 | 2.57E-03 | 0.038 | -3.03E-03 | 2.47E-03 | 0.221 |
| Sex (1=Male) | -3.640 | 0.690 | <.0001 | -3.657 | 0.642 | <.0001 | -2.687 | 0.641 | <.0001 |
| # of medical diagnoses | | | | 2.160 | 0.258 | <.0001 | 1.436 | 0.273 | <.0001 |
| Activities of Daily Living | | | | | | | 0.235 | 0.131 | 0.073 |
| Health Belief Score | | | | | | | 0.687 | 0.262 | 0.009 |
| Physical Pain | | | | | | | -0.002 | 0.611 | 0.998 |
| Problems with Activities | | | | | | | -2.487 | 0.820 | 0.003 |
| Can Walk All Day | | | | | | | -1.365 | 0.678 | 0.045 |
| Problems Seeing | | | | | | | 1.362 | 0.281 | <.0001 |
| Problems Hearing | | | | | | | -0.426 | 0.340 | 0.211 |

**Table S10. Tsimane' depressed affect score OLS regression models.** Data restricted to those with complete data for Model 3 (n=478). From baseline to full model**,** the percentage reduction in depression score due to age alone from ages 20 to 70 is 41.2% lower after controlling for health-related factors.

| Predictors | Model 1 b | Model 1 p | Model 2 b | Model 2 p | Model 3 b | Model 3 p | Model 4 b | Model 4 p |
|---|---|---|---|---|---|---|---|---|
| Age (y) | 0.042 | 0.663 | 0.795 | 0.238 | 0.786 | 0.307 | 1.036 | 0.299 |
| | (0.097) | | (0.673) | | (0.770) | | (0.995) | |
| $Age^2$ | 1.10E-04 | 0.899 | -7.37E-03 | 0.210 | -7.60E-03 | 0.262 | -1.01E-02 | 0.256 |
| | (8.65E-04) | | (5.87E-03) | | (6.77E-03) | | (8.85E-03) | |
| Sex (1=Male) | -3.179 | <.0001 | 1.090 | 0.417 | 1.571 | 0.306 | -16.869 | <.0001 |
| | (0.349) | | (1.343) | | (1.532) | | (2.264) | |
| Number of Diagnoses | | | | | 0.781 | 0.003 | 0.645 | 0.055 |
| | | | | | (0.259) | | (0.335) | |
| Health Belief Score | | | | | | | 0.992 | 0.023 |
| | | | | | | | (0.434) | |
| Physical Pain | | | | | | | -1.560 | 0.056 |
| | | | | | | | (0.813) | |
| Problems with Activities | | | | | | | -0.973 | 0.342 |
| | | | | | | | (1.022) | |
| Can Walk All Day | | | | | | | -0.192 | 0.835 |
| | | | | | | | (0.923) | |
| Intercept | 37.531 | <.0001 | 26.198 | 0.067 | 25.285 | 0.122 | 16.349 | 0.551 |
| | (2.633) | | (14.266) | | (16.329) | | (27.366) | |
| Fixed effects? | No | | Yes | | Yes | | Yes | |
| $R^2$ | 0.23 | | 0.66 | | 0.73 | | 0.78 | |
| n | 1534 | | 1534 | | 1324 | | 979 | |

**Table S11. Tsimane' longitudinal data, among adults with 2+ data points.** (n=1,804 observations among 685 adults; average time between samples is 2.5 years). Models below use linear age and quadratic $age^2$ terms. Alternative specifications of Models 1-4 with only a linear age term show the following results for age: Model 1: b=0.054, p<0.0001; Model 2: b=-0.007, p=0.958; Model 3: -0.038, p=0.823; Model 4: b=-0.045, p=0.831. Numbers in parentheses are standard errors.

| *When was life better for older adults?* | n (%) |
| --- | --- |
| "Present" | 293 (73.8%) |
| "Past" | 104 (26.2%) |

**Table S12. Tsimane reflections on when life was best for older adults.** Binary responses to the question "Which do you think is better? The lives of older adults ("*isho muntyi*") now [in the "present"] or how their lives used to be before, like many years ago [in the "past"]?" Survey administered to Tsimane adults age 55+ y (n=397) as part of the Tsimane Health and Life History Project from 2022-2023.
